# Spermidine inhibits vascular calcification in chronic kidney disease through modulation of SIRT1 signaling pathway

**DOI:** 10.1111/acel.13377

**Published:** 2021-05-09

**Authors:** Xiaoyu Liu, An Chen, Qingchun Liang, Xiulin Yang, Qianqian Dong, Mingwei Fu, Siyi Wang, Yining Li, Yuanzhi Ye, Zirong Lan, Yanting Chen, Jing‐Song Ou, Pingzhen Yang, Lihe Lu, Jianyun Yan

**Affiliations:** ^1^ Department of Cardiology Laboratory of Heart Center Heart Center Zhujiang Hospital Southern Medical University Guangzhou China; ^2^ Guangdong Provincial Biomedical Engineering Technology Research Center for Cardiovascular Disease Guangzhou China; ^3^ Sino‐Japanese Cooperation Platform for Translational Research in Heart Failure Guangzhou China; ^4^ Department of Anesthesiology The Third Affiliated Hospital Southern Medical University Guangzhou China; ^5^ Department of Pathophysiolgy Zhongshan School of Medicine Sun Yat‐Sen University Guangzhou China; ^6^ Division of Cardiac Surgery The First Affiliated Hospital Sun Yat‐Sen University Guangzhou China

**Keywords:** aging, chronic kidney disease, endoplasmic reticulum stress, SIRT1, spermidine, vascular calcification

## Abstract

Vascular calcification is a common pathologic condition in patients with chronic kidney disease (CKD) and aging individuals. It has been established that vascular calcification is a gene‐regulated biological process resembling osteogenesis involving osteogenic differentiation. However, there is no efficient treatment available for vascular calcification so far. The natural polyamine spermidine has been demonstrated to increase life span and protect against cardiovascular disease. It is unclear whether spermidine supplementation inhibits vascular calcification in CKD. Alizarin red staining and quantification of calcium content showed that spermidine treatment markedly reduced mineral deposition in both rat and human vascular smooth muscle cells (VSMCs) under osteogenic conditions. Additionally, western blot analysis revealed that spermidine treatment inhibited osteogenic differentiation of rat and human VSMCs. Moreover, spermidine treatment remarkably attenuated calcification of rat and human arterial rings ex vivo and aortic calcification in rats with CKD. Furthermore, treatment with spermidine induced the upregulation of Sirtuin 1 (SIRT1) in VSMCs and resulted in the downregulation of endoplasmic reticulum (ER) stress signaling components, such as activating transcription factor 4 (ATF4) and CCAAT/enhancer‐binding protein homologous protein (CHOP). Both pharmacological inhibition of SIRT1 by SIRT1 inhibitor EX527 and knockdown of SIRT1 by siRNA markedly blocked the inhibitory effect of spermidine on VSMC calcification. Consistently, EX527 abrogated the inhibitory effect of spermidine on aortic calcification in CKD rats. We for the first time demonstrate that spermidine alleviates vascular calcification in CKD by upregulating SIRT1 and inhibiting ER stress, and this may develop a promising therapeutic treatment to ameliorate vascular calcification in CKD.

## Graphical abstract

We reported that spermidine (Spd) inhibits calcification of vascular smooth muscle cells, arterial rings and aortas in rats with chronic kidney disease (CKD). Furthermore, SIRT1 signal is critical for the inhibitory role of Spd in vascular calcification. Taken together, these findings provide the first evidence that Spd inhibits arterial calcification through modulation of SIRT1 and ER stress signals, suggesting that Spd may act as a novel SIRT1 modulator to treat arterial calcification.

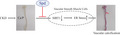

**Abbreviations**
ALP: alkaline phosphatase
ATF4: activating transcription factor 4
BMP2: bone morphogenetic protein‐2
CHOP: CCAAT/enhancer‐binding protein homologous protein
CKD: chronic kidney disease
CM: calcifying medium
DMEM: Dulbecco's modified eagle's medium
ER: endoplasmic reticulum
FBS: fetal bovine serum
GM: growth medium
Runx2: runt‐related transcription factor 2
SIRT1: sirtuin 1
Spd: spermidine
VSMCs: vascular smooth muscle cells

## 1 INTRODUCTION

Vascular calcification refers to the aberrant calcium phosphate crystal deposition in the vessel wall (McCullough et al., 2008), which is a common pathologic condition in patients with chronic kidney disease (CKD), cardiovascular disease, diabetes, and aging individuals (Shroff et al., 2013). A large number of recent studies have suggested that vascular calcification is a gene‐regulated biological process resembling bone mineralization involving osteogenic differentiation (Kong et al., 2018; Lee et al., 2019; Li et al., 2019). Vascular calcification is a common pathological feature of vascular injury such as atherosclerosis and vascular aging. Risk factors of vascular injury such as inflammation, oxidative stress, and calcium and phosphate imbalance have been demonstrated to trigger the development of vascular calcification (Al‐Aly, 2011; Aikawa et al., 2007; Chen et al., 2020; Durham et al., 2018). Inhibition of osteogenic differentiation of vascular smooth muscle cells may represent a promising strategy to prevent or reverse vascular calcification.

Vascular calcification is a characteristic of vascular stiffness and aging. Accumulating evidence has indicated that aging is an important contributor to vascular calcification (Pescatore et al., 2019). Senescent vascular smooth muscle cells positively regulate vascular calcification through increasing osteogenic transition. Sirtuin 1 (SIRT1) is a nicotinamide adenine dinucleotide‐dependent histone deacetylase regulating cell cycle, differentiation, apoptosis, and metabolism (Bordone & Guarente, 2005; Buhrmann et al., 2014; Gorenne et al., 2013; Hsu et al., 2017). SIRT1 plays critical roles in age‐related pathological changes such as cardiovascular disease, neurodegenerative disease, and metabolic diseases (Zeng et al., 2009). A number of studies have shown that SIRT1 inhibits senescence of vascular smooth muscle cells and endothelial cells (Ota et al., 2007; Takemura et al., 2011; Wan et al., 2014). In addition, SIRT1 is an important factor and attractive candidate regulating vascular calcification (Bartoli‐Leonard et al., 2018). Several in vitro studies have reported that SIRT1 exerts a protective role in vascular calcification (Bartoli‐Leonard et al., 2019; Takemura et al., 2011). Moreover, animal studies have demonstrated that resveratrol, a commonly used activator of SIRT1, attenuates vascular calcification in both in uremic mice and non‐human primates (Mattison et al., 2014; Tomayko et al., 2014). In contrast, suppression of SIRT1 promotes vascular calcification (Bartoli‐Leonard et al., 2021). Therefore, development of SIRT1 modulators may provide a potential strategy to inhibit vascular calcification.

Spermidine (Spd), a naturally synthesized polyamine, has attracted increased attention because of its capability of extending life span and protecting against cardiovascular disease (Eisenberg et al., 2016). Vascular calcification is thought to be a common hallmark of advanced stage of atherosclerosis. It has been reported that Spd reduces lipid accumulation to retard the progression of atherosclerosis (Michiels et al., 2016). In addition, Spd ameliorates cardiomyocyte aging by activating the SIRT1 signaling pathway (Wang et al., 2020). SIRT1 is also required for Spd‐induced life‐span extension. Our recent study has shown that Spd inhibits myocardial apoptosis, improves cardiac dysfunction, and attenuates myocardial injury (Yan et al., 2019). Nevertheless, the role of Spd in osteogenic differentiation of vascular smooth muscle cells and arterial calcification is currently unknown. The link between Spd and SIRT1 during the process of vascular calcification has not been reported before. In this study, using in vitro, ex vivo, and in vivo models of vascular calcification, we investigated whether Spd treatment inhibits vascular calcification.

## 2 RESULTS

### 2.1 Spd attenuates calcification of rat and human vascular smooth muscle cells

**FIGURE 1:**
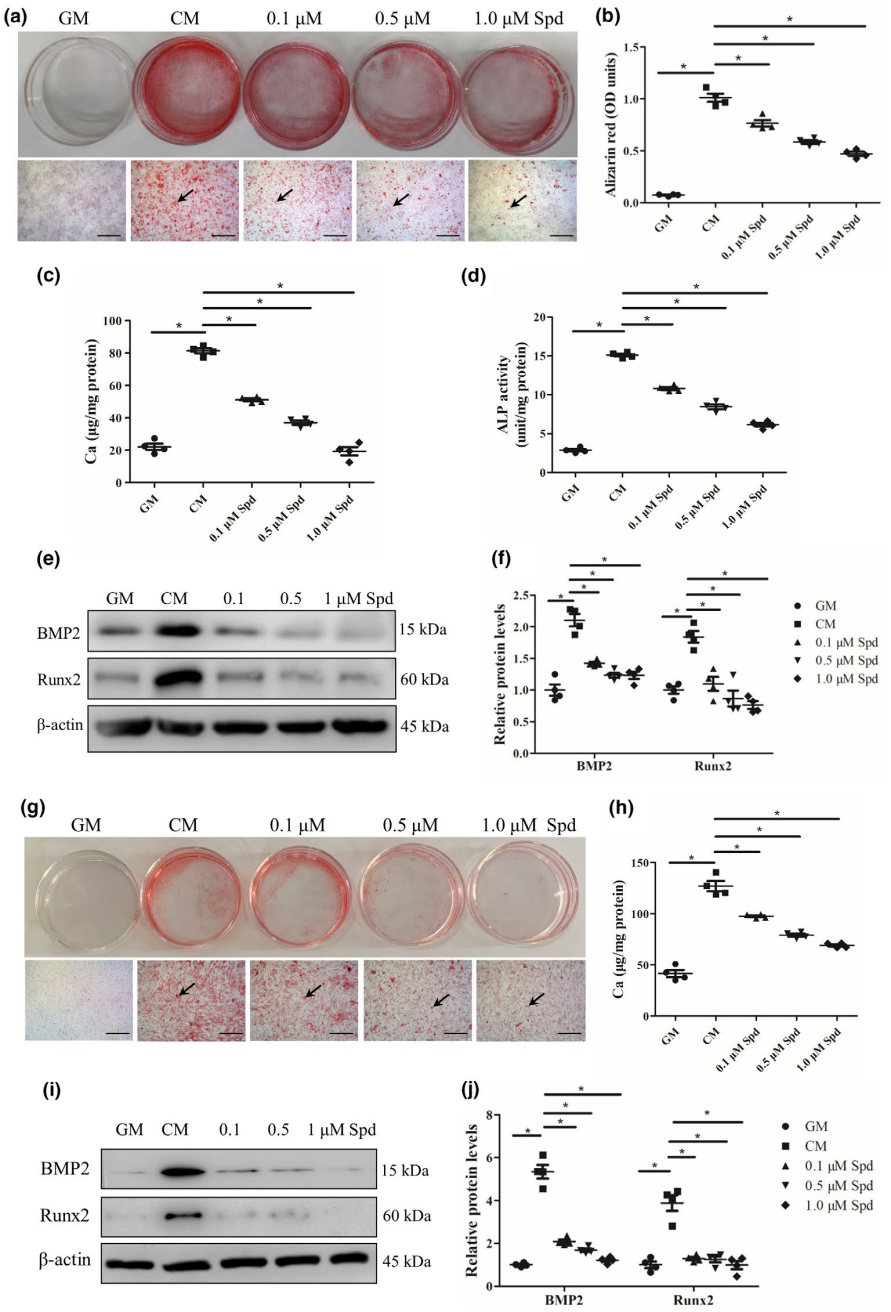
Spd inhibits rat and human vascular smooth muscle cell calcification. Rat (a‐f) and human (g‐j) vascular smooth muscle cells were incubated with growth medium (GM), or calcifying medium (CM) with or without Spd (0.1, 0.5, 1.0 μM) for 7 days (*n *= 4). (a, g) Alizarin red staining was used to assess cell calcification. Representative images showing cells stained with alizarin red solution. Scale bar = 500 µm. (b) Quantitative analysis of alizarin red staining by a microplate reader. (c, h) Quantitative analysis of calcium content by a Ca assay kit. (d) Quantification of ALP activity. (e, i) Representative western blots for BMP2 and Runx2. (f, j) Quantification of BMP2 and Runx2 by densitometry. *p < 0.01. One‐way ANOVA, Tukey's HSD post hoc test

To investigate whether Spd treatment affects the progression of vascular calcification in vitro, rat vascular smooth muscle cells were grown in calcifying medium (CM) containing Spd (0.1, 0.5, and 1 μM). Of note, alizarin red staining revealed that calcification was observed in CM‐treated cells, but not in cells maintained in growth medium (GM), suggesting that vascular smooth muscle cell calcification was successfully induced under osteogenic conditions. Interestingly, vascular smooth muscle cells treated with Spd showed reduced calcification compared to CM‐treated cells at day 7 (Figure 1a), and this was further confirmed by quantification analysis of alizarin red staining (*p *< 0.01, Figure 1b). In addition, calcium content assay showed that Spd treatment significantly reduced calcium content (*p *< 0.01, Figure 1c). Furthermore, we performed alkaline phosphatase (ALP) activity assay and western blot analysis to examine the effect of Spd on osteogenic differentiation of vascular smooth muscle cells. Consistently, the activity of ALP, an osteogenic differentiation marker, was reduced in Spd‐treated cells compared to CM‐treated cells at day 7 (*p* < 0.01, Figure 1d). We also found that the expression of other osteogenic differentiation markers such as bone morphogenetic protein‐2 (BMP2) and Runt‐related transcription factor 2 (Runx2) was significantly downregulated in vascular smooth muscle cells treated with Spd compared with untreated controls at day 7 (*p* < 0.01, Figure 1e,f), indicating that Spd repressed osteogenic differentiation of vascular smooth muscle cells. Next, we examined the effect of Spd (1 μM) on rat vascular smooth muscle cell calcification at different time points. Similarly, a time‐course experiment also showed that Spd inhibited mineralization in rat vascular smooth muscle cells under osteogenic conditions (Figure S1a). Quantification analysis of alizarin red staining further demonstrated the protective role of Spd in the progression of rat vascular smooth muscle cell calcification (*p* < 0.01, Figure S1b).

To validate the inhibitory effect of Spd on vascular smooth muscle cell calcification, we next treated human vascular smooth muscle cells with CM supplemented with Spd (0.1, 0.5, and 1 μM). As indicated by alizarin red staining, Spd treatment dose‐dependently decreased calcification of human vascular smooth muscle cells under osteogenic conditions at day 7 (Figure 1g). This inhibitory effect of Spd on calcification in human vascular smooth muscle cells was confirmed by quantification analysis of alizarin red staining (*p* < 0.01, Figure S2). Additionally, lower calcium levels were found in Spd‐treated cells than CM‐treated cells (*p* < 0.01, Figure 1h). Moreover, western blots showed Spd‐induced down‐regulation of BMP2 and Runx2 in human vascular smooth muscle cells at day 7 (Figure 1i,j). Collectively, these data demonstrate that Spd inhibits the osteogenic differentiation and mineralization of vascular smooth muscle cells under osteogenic conditions.

### 2.2 Spd inhibits senescence of rat vascular smooth muscle cells

Next, we examined effect of Spd on vascular smooth muscle cell senescence. SA‐beta‐gal staining revealed that osteogenic condition stimulated rat vascular smooth muscle cell senescence at day 7, and this effect was inhibited by Spd treatment (Figure S3a). In addition, CM‐treated cells showed increased p21 expression compared to GM‐treated cells at day 7 and this was blocked by Spd treatment (Figure S3b). Quantification analysis further confirmed the inhibitory role of Spd in regulation of p21 expression (*p* < 0.01, Figure S3c). These findings suggest that Spd inhibits vascular smooth muscle cell senescence.

### 2.3 Spd attenuates calcification of rat and human arterial rings

**FIGURE 2:**
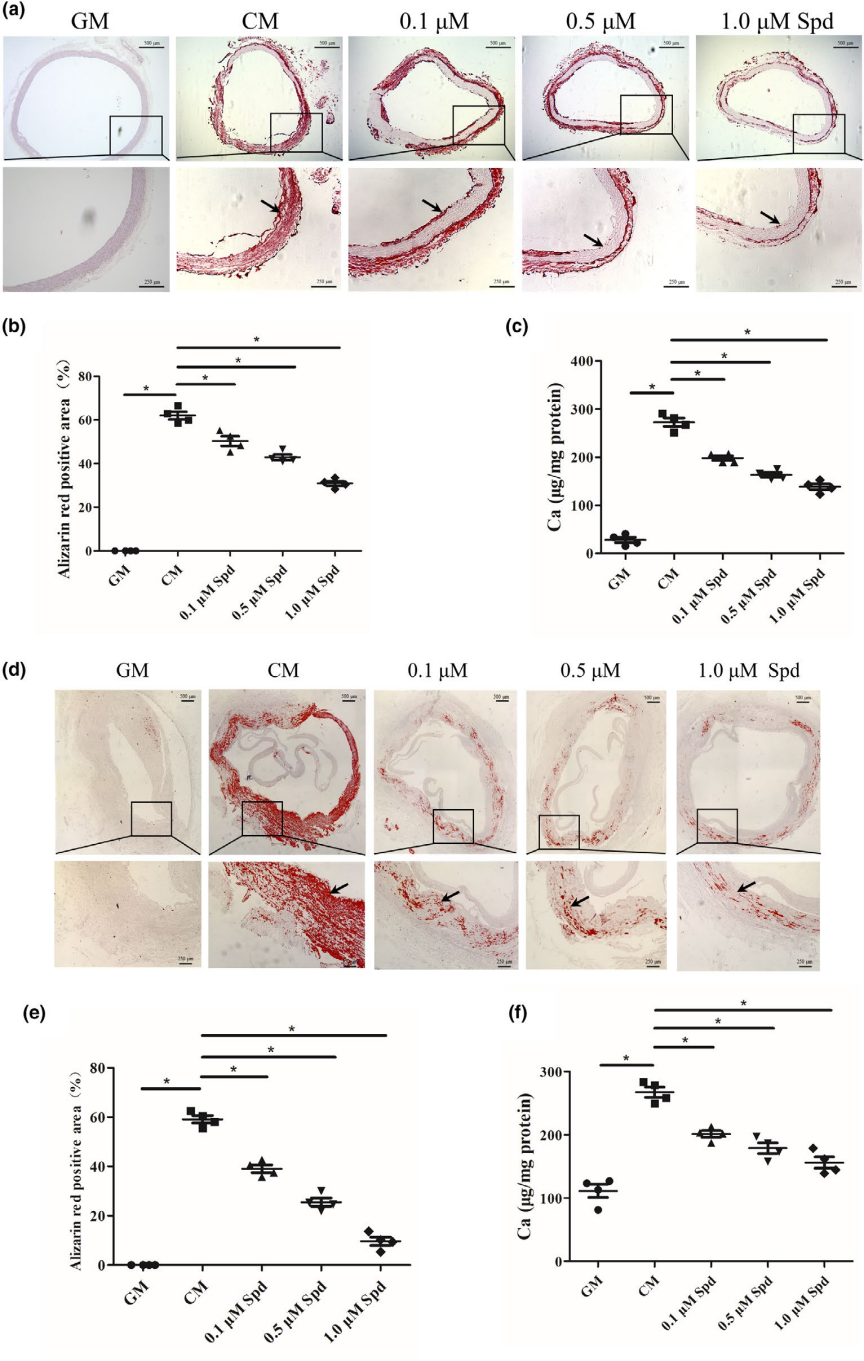
Spd inhibits calcification of rat and human arterial rings. Rat aortic rings (a‐c) and human arterial rings (d‐f) were incubated with growth medium (GM), or calcifying medium (CM) with or without Spd (0.1, 0.5, 1.0 μM) for 7 days (*n *= 4). (a, d) Alizarin red staining of arteries for detecting calcification. Representative images showing arteries stained with alizarin red solution. Scale bar = 500 µm (upper) and 250 µm (lower). (b, e) Quantitative analysis of alizarin red staining by a microplate reader. (c, f) Quantitative analysis of calcium content by a Ca assay kit. *p < 0.01. One‐way ANOVA, Tukey's HSD post hoc test

To investigate whether Spd treatment has an effect on vascular calcification ex vivo, rat aortic rings and human arterial rings were incubated with calcifying medium containing different concentrations of Spd (0.1, 0.5, and 1 μM). Notably, treatment of rat aortic arteries with Spd decreased vascular calcification at day 7, as indicated by alizarin red staining (*p* < 0.01, Figure 2a,b). Additionally, we found that calcium content was significantly decreased in Spd‐treated rat aortic rings compared to untreated controls (*p* < 0.01, Figure 2c). Consistently, alizarin red staining revealed that Spd also reduced mineral deposition in human arteries (Figure 2d,e). Furthermore, calcium content analysis verified the inhibitory effect of Spd on mineralization in human arteries (*p* < 0.01, Figure 2f). Taken together, these findings suggest that Spd attenuates calcification of rat and human arterial rings ex vivo.

### 2.4 Spd attenuates aortic calcification in CKD rats

**FIGURE 3:**
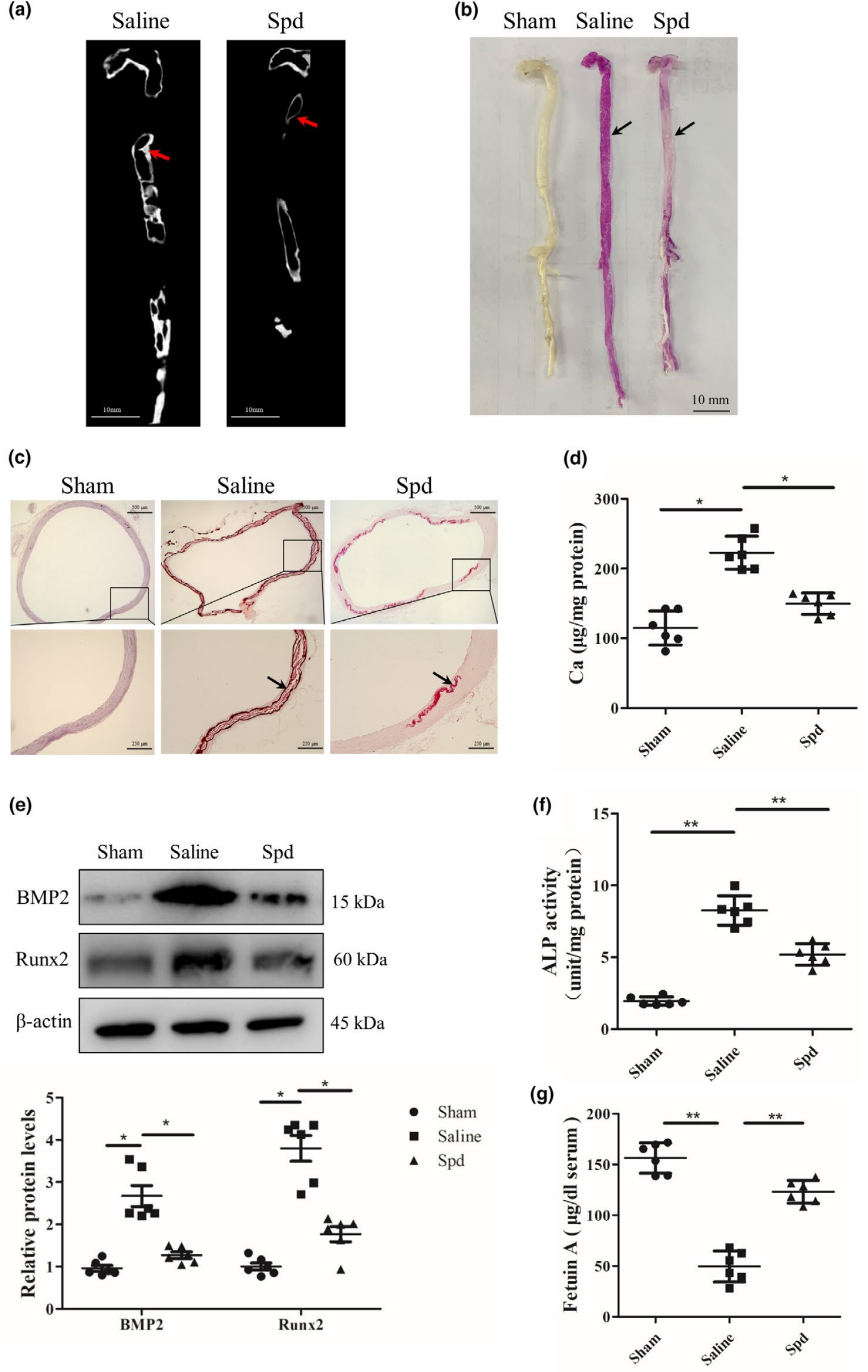
Spd inhibits aortic calcification in CKD rats. Rats subjected to surgery were treated with Spd (3 mM) in drinking water for 4 weeks (*n *= 6). (a) Aortic calcification (red arrow) was detected by micro‐CT. (b, c) Mineral deposition in aortic arteries and sections was assessed by alizarin red staining solution. Representative images showing aortic arteries and sections stained with alizarin red. Scale bar = 500 µm (upper) and 250 µm (lower). (d) Calcium content of aortas was measured. One‐way ANOVA, Tukey's HSD post hoc test. (e) Representative western blots for BMP2 and Runx2 protein expression and quantification of BMP2 and Runx2 by densitometry. Tamhane T2 test. (f) Quantification of ALP activity. (g) Quantification of fetuin‐A levels. *p < 0.05, **p < 0.01. One‐way ANOVA, Tukey's HSD post hoc test

It is well known that vascular calcification is commonly found in CKD patients (Yamada & Giachelli, 2017; O'Neill & Lomashvili, 2010). We next tested whether Spd supplementation inhibits aortic calcification in CKD rats. Rats were randomly assigned into sham group, model (saline) group, and Spd group. Rats from CKD group and Spd group were subjected to 5/6 nephrectomy, while rats from sham group underwent sham operation. We measured blood creatinine levels to evaluate the development of rat CKD model 2 weeks after surgery. Compared to sham controls, creatinine levels were highly increased in rats from model group and Spd group (*p* < 0.01, Figure S4). We next performed Micro‐CT analysis and alizarin red staining to examine the extent of aortic calcification. Of interest, Micro‐CT analysis showed that Spd‐treated rats displayed a reduced calcification of the aortas compared to saline‐treated rats (Figure 3a). As expected, alizarin red staining of aortas also revealed that Spd treatment reduced aortic calcification (Figure 3b) and this result was further confirmed by alizarin red staining of aortic sections (Figure 3c). Moreover, we found that calcium content in aortic arteries was significantly decreased after treatment with Spd (*p* < 0.05, Figure 3d). Furthermore, we examined the influence of Spd on osteogenic differentiation using western blot analysis. The results demonstrated that Spd treatment inhibited osteogenic differentiation, as evidenced by downregulation of BMP2 and Runx2 in aortic arteries (*p* < 0.05, Figure 3e), and reduced ALP activity (*p* < 0.01, Figure 3f). Additionally, we found that saline‐treated rats displayed a reduced levels of fetuin‐A, an inhibitor of vascular calcification, compared with sham controls, and Spd treatment increased serum levels of fetuin‐A in CKD rats (*p* < 0.01, Figure 3g).

### 2.5 SIRT1 signal mediates vascular smooth muscle cell calcification inhibition by Spd

**FIGURE 4:**
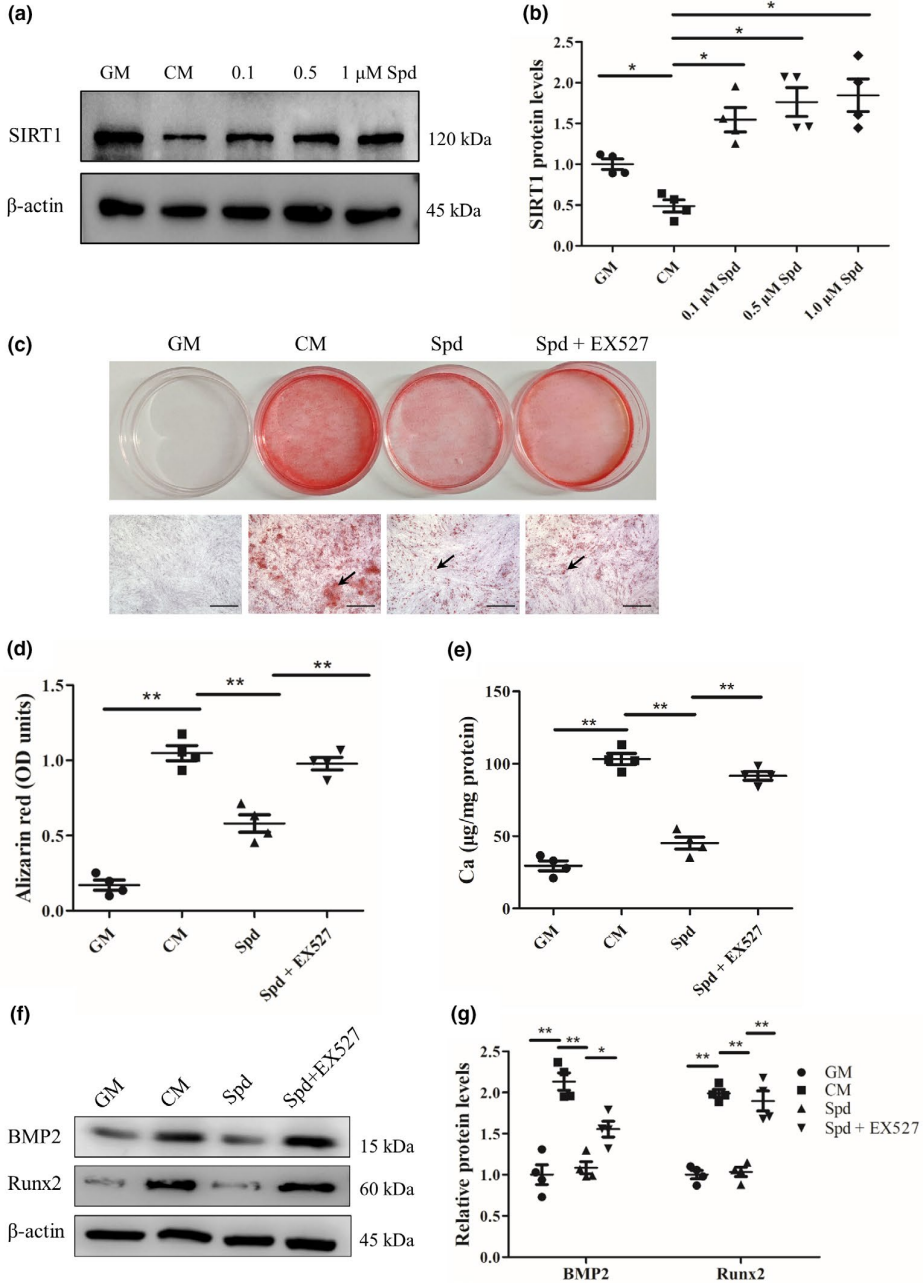
Pharmacological inhibition of SIRT1 by EX527 counteracted the protective effect of Spd on vascular smooth muscle cell calcification. Rat vascular smooth muscle cells were incubated with Spd (0.1, 0.5, 1.0 μM) together with calcifying medium (CM), CM alone or growth medium (GM) for 7 days (*n *= 4). (a) Representative western blots for SIRT1. (b) Quantification of SIRT1 by densitometry. Tamhane T2 test. Rat vascular smooth muscle cells were treated with Spd (1.0 μM) together with EX527 (10 μM) in the presence of CM for 7 days. (c) Representative images showing cells stained with alizarin red solution. Scale bar=500 µm. (d) Quantitative analysis of alizarin red staining by a microplate reader. (e) Quantitative analysis of calcium content by a Ca assay kit. (f) Representative western blots for BMP2 and Runx2 protein expression. (g) Quantification of BMP2 and Runx2 by densitometry. *p < 0.05; **p < 0.01. (d), (e) and (g) using one‐way ANOVA, Tukey's HSD post hoc test

It has been reported that upregulation of SIRT1 can be induced by Spd and SIRT1 inhibits vascular calcification (Wang et al., 2020; Takemura et al., 2011). Therefore, we examined whether Spd affects SIRT1 expression in vascular smooth muscle cells. Spd at different concentrations (0.1, 0.5, and 1 μM) was used to treat rat vascular smooth muscle cells under osteogenic conditions. Western blots revealed that SIRT1 expression was highly increased in Spd‐treated cells compared with CM‐treated cells (*p* < 0.05, Figure 4a,b). These results suggest a potential role of SIRT1 in regulation of Spd‐mediated inhibition of vascular smooth muscle cell calcification. To determine whether SIRT1 signal is required for the inhibitory role of Spd in vascular calcification, we incubated rat vascular smooth muscle cells with SIRT1 inhibitor, EX527 (10 μM). Of interest, alizarin red staining demonstrated that Spd treatment markedly reduced vascular smooth muscle cell calcification and this effect was blocked by EX527 (Figure 4c). Moreover, quantification analysis of alizarin red staining revealed that SIRT1 inhibition by EX527 abrogated the inhibitory role of Spd in vascular calcification (*p* < 0.01, Figure 4d), and this result was verified by calcium content assay (*p* < 0.01, Figure 4e). In addition, we found that EX527 prevented Spd‐induced downregulation of BMP2 and Runx2 in vascular smooth muscle cells (Figure 4f,g).

**FIGURE 5:**
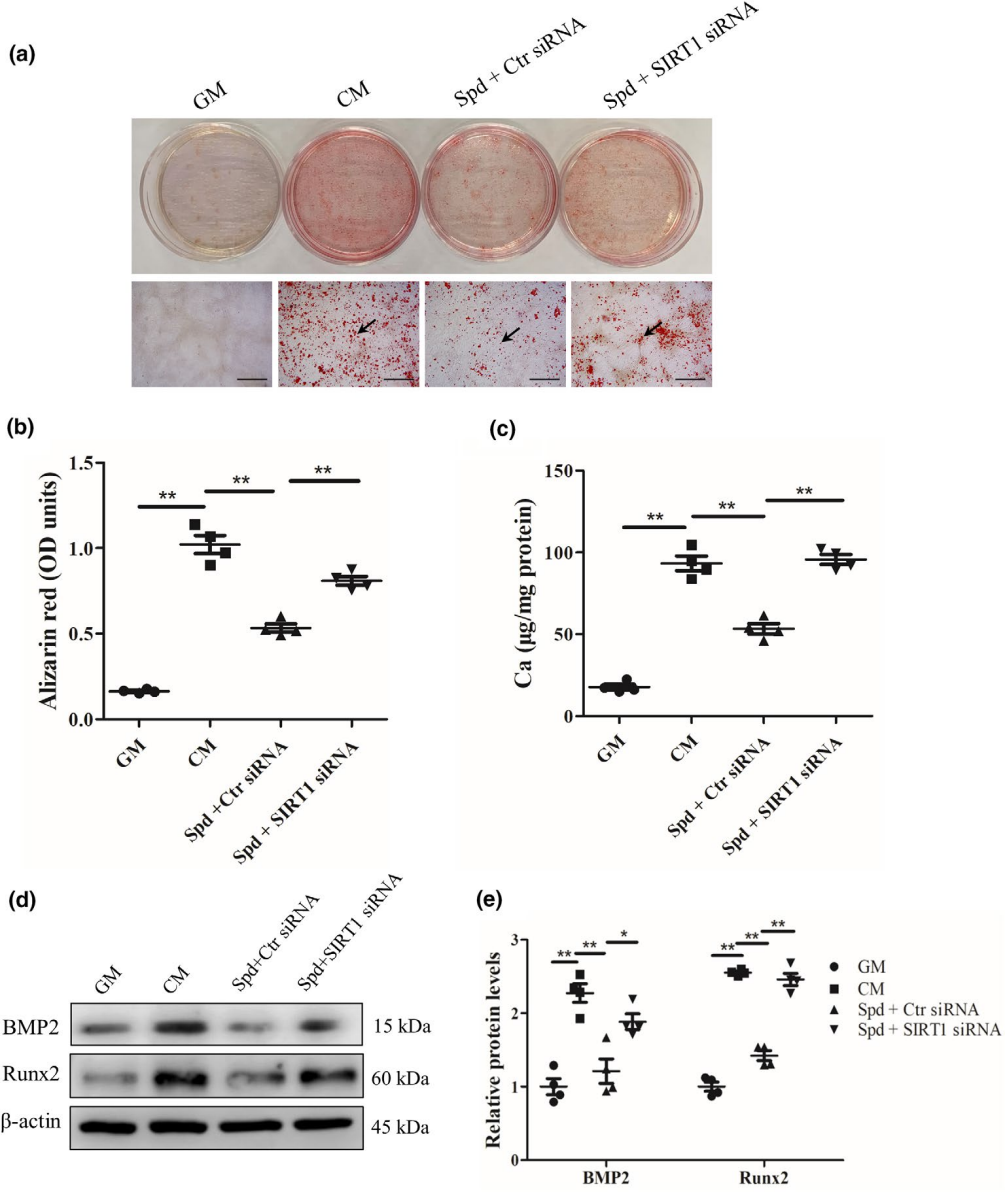
Knockdown of SIRT1 by siRNA abrogated the protective role of Spd in vascular smooth muscle cell calcification. Rat vascular smooth muscle cells were exposed to growth medium (GM), calcifying medium (CM), CM containing Spd (1.0 μM) together with control (Ctr) siRNA, or CM containing Spd (1.0 μM) together with SIRT1 siRNA (15 nM) for 7 days. (a) Representative images showing cells stained with alizarin red solution. Scale bar = 500 µm. (b) Quantitative analysis of alizarin red staining by a microplate reader. Tamhane T2 test. (c) Quantitative analysis of calcium content by a Ca assay kit. (d) Representative western blots for BMP2 and Runx2 protein expression. (e) Quantification of BMP2 and Runx2 by densitometry. *p < 0.05; **p < 0.01. One‐way ANOVA, Tukey's HSD post hoc test

To validate the requirement of SIRT1 signal for the inhibitory role of Spd in vascular calcification, we next examined the effect of SIRT1 siRNA on vascular calcification in the presence of Spd. Western blot analysis showed that SIRT1 protein expression in vascular smooth muscle cells transfected with SIRT1 siRNA was greatly downregulated compared to cells transfected with control siRNA (Figure S5a), suggesting the successful knockdown of SIRT1 by siRNA in vascular smooth muscle cells. Alizarin red staining showed that SIRT1 siRNA markedly blocked the inhibitory effect of Spd on vascular smooth muscle cell calcification (Figure 5a). Similarly, quantification analysis showed that SIRT1 siRNA significantly abrogated the inhibitory effect of Spd on vascular smooth muscle cell calcification (*p* < 0.01, Figure 5b,c). Furthermore, western blot analysis revealed that Spd‐induced decrease in BMP2 and Runx2 expression levels in vascular smooth muscle cells was also inhibited by SIRT1 siRNA (Figure 5d,e). Hence, these results indicate that SIRT1 signal plays a critical role in inhibitory regulation of vascular smooth muscle cell calcification by Spd treatment.

### 2.6 Spd attenuates in vivo aortic calcification in CKD rats involving SIRT1 signal

**FIGURE 6:**
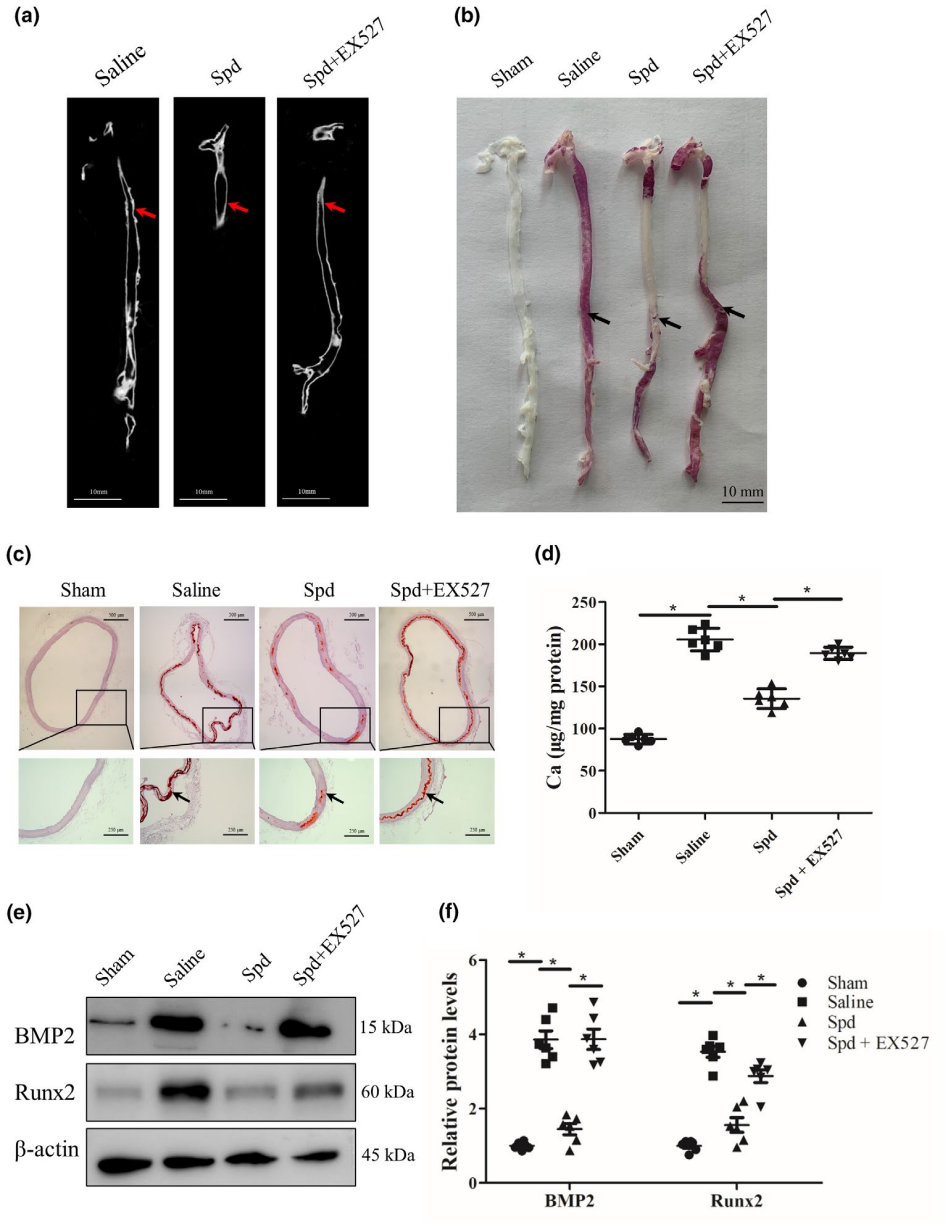
Effect of SIRT1 inhibition by EX527 on aortic calcification in rats with chronic kidney disease. Rats subjected to surgery were treated with Spd (3 mM) in drinking water together with EX527 (5 mg/kg, i.p.) for 4 weeks (*n *= 6). (a) Aortic calcification (red arrow) was detected by micro‐CT. (b, c) Mineral deposition in aortic arteries and sections was assessed by alizarin red staining. Representative images showing aortic arteries and sections stained with alizarin red solution. Scale bar = 500 µm (upper) and 250 µm (lower). (d) Calcium content of aortas was measured. (e) Representative western blots for BMP2 and Runx2 protein expression. (f) Quantification of BMP2 and Runx2 by densitometry. *p < 0.05. One‐way ANOVA, Tukey's HSD post hoc test

To further confirm whether SIRT1 signal is required for Spd‐mediated inhibition of vascular calcification in vivo, we treated CKD rats with Spd together with SIRT1 inhibitor, EX527. Micro‐CT analysis revealed less aortic calcification in Spd‐treated rats than that in saline‐treated rats, and the inhibitory effect by Spd was abrogated by EX527 (Figure 6a). Next, we performed alizarin red staining to examine aortic calcification. Of note, whole mount staining of aortas revealed that Spd‐treated rats displayed reduced aortic calcification compared to saline‐treated rats, and SIRT1 inhibition by EX527 abolished the protective effect of Spd on aortic calcification (Figure 6b). Staining of aortic sections further demonstrated Spd treatment reduced aortic calcification, which was also counteracted by EX527 (Figure 6c). Moreover, calcium content in aortas, measured by calcium content assay, was significantly reduced upon Spd treatment and this reduction induced by Spd was prevented by EX527 (*p* < 0.05, Figure 6d). Furthermore, we observed that aortic expression of BMP2 and Runx2 in rats treated with Spd was significantly decreased compared with rats treated with saline, but was increased in Spd together with EX527‐treated rats compared with Spd‐treated rats (*p* < 0.01, Figure 6e,f). Collectively, these findings indicate that SIRT1 signal is critically important for Spd‐mediated inhibitory effect on arterial calcification.

### 2.7 Inhibition of ER stress is required for inhibition of vascular smooth muscle cell calcification by Spd

**FIGURE 7:**
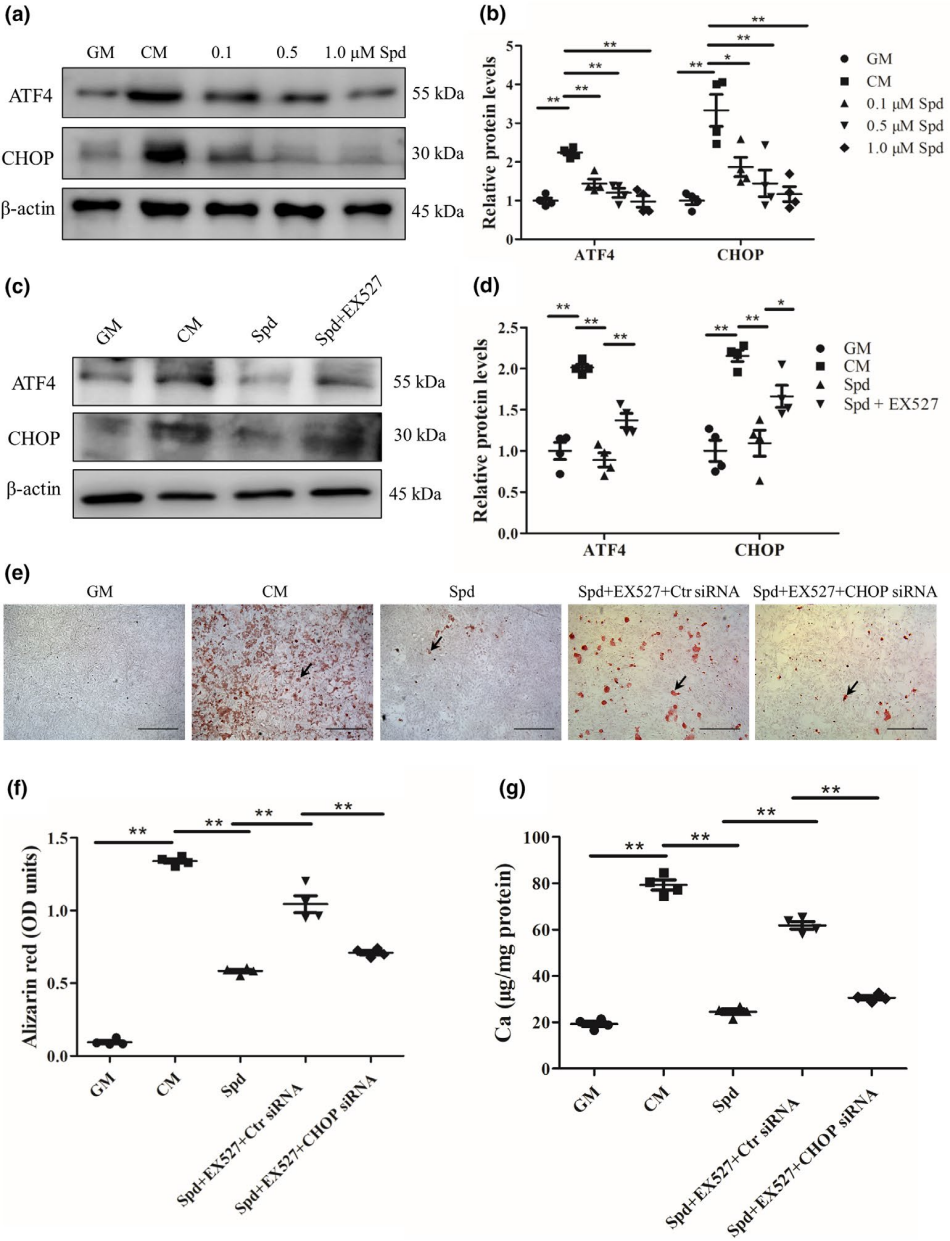
Spd inhibits vascular smooth muscle cell calcification through SIRT1‐mediated inhibition of ER stress. Rat vascular smooth muscle cells were incubated with growth medium (GM), or calcifying medium (CM) with or without different concentrations of Spd (0.1, 0.5, 1.0 μM) for 7 days (*n *= 4). (a) Representative western blots for ATF4 and CHOP protein expression. (b) Quantification of ATF4 and CHOP by densitometry. Rat vascular smooth muscle cells were incubated with GM, CM, CM containing Spd (1.0 μM), or CM containing Spd (1.0 μM) together with EX527 (10 μM) for 7 days. (c) Representative western blots for ATF4 and CHOP. (d) Quantification of ATF4 and CHOP by densitometry. Rat vascular smooth muscle cells were transfected with CHOP siRNA (30 nM) or control siRNA in the presence of CM containing Spd (1.0 μM) and EX527 (10 μM) for 7 days. (e) Alizarin red staining was used to detect cell calcification. Representative images showing cells stained with alizarin red solution. Scale bar = 500 µm. (f) Quantitative analysis of alizarin red staining by a microplate reader. Tamhane T2 test. (g) Quantitative analysis of calcium content by a Ca assay kit. *p < 0.05; **p < 0.01. B, D and G using one‐way ANOVA, Tukey's HSD post hoc test

Previous studies have shown that endoplasmic reticulum (ER) stress plays critical roles in regulating vascular calcification (Shanahan & Furmanik, 2017; Masuda et al., 2016). To further understand the mechanism by Spd inhibits vascular calcification, we carried out western blot analysis to test whether Spd treatment impacts ER stress in vascular smooth muscle cells. Interestingly, Spd significantly reduced the expression of ATF4 and CHOP in rat vascular smooth muscle cells at day 7 (Figure 7a,b), but the downregulation of ATF4 and CHOP was reversed by SIRT1 inhibitor EX527 (Figure 7c,d), suggesting that Spd inhibited ER stress through activation of SIRT1. We next treated rat vascular smooth muscle cells with Spd together with EX527 and CHOP siRNA. Western blot analysis confirmed the successful knockdown of CHOP by siRNA in vascular smooth muscle cells (Figure S5b). As shown in Figure 7e,f, Spd treatment resulted in reduced calcification of vascular smooth muscle cells and the inhibitory effect was prevented by EX527. Additionally, CHOP siRNA counteracted the effect of EX527 on calcification of vascular smooth muscle cells (*p* < 0.01). Furthermore, lower calcium levels in CHOP siRNA‐treated cells were found than control siRNA‐treated cells in the presence of Spd and EX527 under osteogenic conditions (*p* < 0.01, Figure 7g). All of these results indicate that Spd treatment ameliorates vascular smooth muscle cell calcification via SIRT1‐mediated inhibition of ER stress.

## 3 DISCUSSION

Vascular calcification occurs frequently in CKD, accompanied by displaying the features of vascular aging (Shanahan, 2013). Currently, no effective therapy is available for vascular calcification due to lack of fully understanding the mechanisms underlying vascular calcification. In this present study, we investigated whether Spd, a natural polyamine, attenuates vascular calcification in CKD. Of interest, we demonstrated that Spd attenuates calcification of vascular smooth muscle cells and arterial rings using cell and tissue culture models. Furthermore, animal study also revealed that Spd treatment ameliorates aortic calcification in CKD rats. In addition, we found that SIRT1 signal is crucial for the inhibitory role of Spd in vascular calcification. To our knowledge, these results provide the first evidence that Spd treatment attenuates arterial calcification in CKD through modulation of SIRT1 and ER stress signals.

Arterial calcification is frequently found in CKD patients and the underlying mechanism remains elusive. Accumulating studies have suggested vascular calcification as a gene‐regulated pathological process sharing the characteristics with bone formation (Tani et al., 2020; Paloian et al., 2016). Therefore, it is possible that vascular calcification could be prevented or reversed by targeting genes implicated in the regulation of vascular calcification. Vascular calcification commonly occurs with aging in the cardiovascular system (Kovacic et al., 2011). Previous studies have shown that genetically extending life span, and eliminating atherosclerosis and ectopic calcification significantly improve premature aging‐like features (Razzaque et al., 2006). Vascular calcification and aging share some common mechanisms (Pescatore et al., 2019). Therefore, anti‐aging reagents could also inhibit vascular calcification. It has been demonstrated that resveratrol attenuates both vascular calcification and vascular aging (Tomayko et al., 2014; Kim et al., 2018). Spd has been demonstrated to preserve mitochondrial function, and prevent stem cell senescence (Madeo et al., 2018), and has emerged as exhibiting anti‐aging properties and cardiovascular protection (Minois, 2014). Since Spd has been shown to extend life span and protect against cardiovascular disease, we speculate that Spd could inhibit vascular calcification. Interestingly, we found that Spd treatment inhibits calcification of rat and human vascular smooth muscle cells. Moreover, Spd treatment remarkably attenuated calcification of rat and human arterial rings ex vivo and aortic calcification in rats with CKD, suggesting that Spd could become an efficient candidate for the treatment of vascular calcification. In addition, a recent study has shown that Spd treatment reduces aortic remodeling and inhibits the development of abdominal aortic aneurysm in mice (Liu et al., 2020). Since Spd exists in the daily diet and has low toxicity yet strong efficacy (Madeo et al., 2018), it might be a promising therapeutic agent for vascular diseases in clinical applications.

SIRT1 has been shown to inhibit vascular calcification (Akiyoshi et al., 2016; Takemura et al., 2011). SIRT1 expression is decreased during vascular calcification, and activation of SIRT1 reduces aging‐associated vascular calcification (Badi et al., 2018). Activation of SIRT1 may represent a promising strategy for the treatment of vascular calcification. Resveratrol, which has a similar biological activity with spermidine (Morselli et al., 2009), has been shown to activate SIRT1 in several studies (Borra et al., 2005; Howitz et al., 2003). It was reported that resveratrol ameliorated vascular calcification by regulating SIRT1 (Zhang et al., 2016). Of interest, we also found that Spd treatment stimulated upregulation of SIRT1 in vascular smooth muscle cells. Furthermore, we observed that both pharmacological inhibition of SIRT1 and knockdown of SIRT1 blocked the inhibitory effect of Spd on vascular smooth muscle cell calcification. Consistently, our animal study confirmed that EX527 abrogated the inhibitory effect of spermidine on aortic calcification in CKD. Altogether, these data support the conclusion that SIRT1 signal is critical for the protective role of Spd in the progression of vascular calcification.

Several studies have established that ER stress plays important roles in the regulation of phenotypic change of vascular smooth muscle cells and vascular calcification (Dong et al., 2020; Duan et al., 2009; Shanahan, 2017). ER stress signaling ATF4/CHOP is activated during vascular calcification (Masuda et al., 2013). Knockdown of CHOP inhibits vascular calcification (Miyazaki‐Anzai et al., 2014). Previous studies have suggested that ER stress response can be modulated by SIRT1 signal (Prola et al., 2017; Kang et al., 2018). Therefore, we examined the effect of SIRT1 signal on ER stress during vascular calcification. Notably, Spd treatment significantly downregulated ATF4 and CHOP in vascular smooth muscle cells, whereas usage of SIRT1 inhibitor EX527 prevented the downregulation of ATF4 and CHOP, suggesting Spd inhibited ER stress through activation of SIRT1. Additionally, the inhibitory effect of Spd on vascular smooth muscle cell calcification was counteracted by EX527 and CHOP siRNA, respectively. Collectively, these findings provide the first evidence that Spd treatment inhibits vascular calcification via SIRT1‐mediated inhibition of ER stress.

The limitation of this study is that Spd exhibits multiple effects including anti‐inflammatory properties, antioxidant functions, and autophagy induction (Minois, 2014), but we only focused on the effect of Spd on SIRT1 and ER stress signals. Therefore, we cannot rule out the possibility that Spd supplementation inhibits vascular calcification through targeting inflammation, oxidative stress, and autophagy. It will be worth investigating whether Spd also affects markers and signaling pathways of inflammation, oxidative stress, and autophagy during vascular calcification in the future. In addition, additional experiment is required to determine the effect of Spd on arterial calcification in SIRT1‐deficienct mice.

In conclusion, our study suggests that Spd acts as a novel regulator of vascular calcification. Moreover, Spd protects against vascular calcification in CKD by modulation of SIRT1 and ER stress signals. Dietary polyamine uptake may serve as a promising strategy for the prevention of vascular calcification. This study paves the way for prospective clinical trials to investigate the beneficial effect of Spd on arterial calcification in CKD patients.

## 4 EXPERIMENTAL PROCEDURES

### 4.1 Animal studies

All animal experiments were approved by the Institutional Animal Care Committee at Zhujiang Hospital, Southern Medical University, China (LAEC‐2020‐064) and conducted in accordance with the US National Institutes of Health Guide for the Care and Use of Laboratory Animals (8th Edition, 2011). Male Sprague–Dawley (SD) rats (220–250 g) were supplied by Central Animal Care Facility of Southern Medical University, China. All rats were housed in standard laboratory conditions with a 12‐h light /12‐h dark cycle and had free access to tap water and food. Rats were randomly assigned into three groups (*n *= 6): namely sham group, model (saline) group, and Spd group. Rat CKD model was established by 5/6 nephrectomy as previously described (Li et al., 2019). Briefly, rats were anesthetized with sodium pentobarbital (50 mg/kg) by intraperitoneal injection and underwent a 2/3 right nephrectomy, followed by surgical extirpation of the total left kidney 1 week later. Serum creatinine levels were measured 2 weeks after surgery. To induce vascular calcification, rats were administrated with a high calcium and phosphorus diet containing 4% calcium and 1.8% phosphate (Guangdong Medical Lab Animal Center), together with 1 µg/kg of calcitriol gavage (Aladdin; Zhang et al., 2020). In some experiments, CKD rats were treated with 3 mM of Spd (Sigma) in drinking water (Eisenberg et al., 2016) or EX527 (5 mg/kg body weight; Selleck) by intraperitoneal injection for 4 weeks (Eid et al., 2020).

### 4.2 Cell culture

Primary rat vascular smooth muscle cells were prepared using explant method as described in our previous studies (Hou et al., 2016; Li et al., 2019). Briefly, rats were intraperitoneally euthanized with sodium pentobarbital (150 mg/kg) and thoracic aortas were isolated from 2‐month‐old male SD rats. Aortic segments were then cut into small pieces and grown in Dulbecco's modified Eagle's medium (DMEM; Thermo Fisher Scientific) containing 10% fetal bovine serum (FBS), 100 U/ml penicillin, and 100 mg/ml streptomycin at 37°C in a humidified incubator. Rat vascular smooth muscle cells were migrated from the aortic explants and maintained in growth medium. Human vascular smooth muscle cells were supplied by American type culture collection (ATCC). Cells were grown in DMEM supplemented with 10% FBS. Primary rat vascular smooth muscle cells from passages 3 to 8 were used in this study. Vascular smooth muscle cell calcification was induced by the addition of DMEM containing 10 mM β‐glycerophosphate and 3 mM calcium chloride (calcifying medium) (Zhang et al., 2020; Li et al., 2019). In some experiments, vascular smooth muscle cells were treated with different concentrations of Spd (0.1, 0.5, and 1 μM) in the presence of calcifying medium for 7 days.

### 4.3 Small interfering RNA (siRNA) transfection

Rat vascular smooth muscle cells were seeded in a 6‐well plate at a density of 2 × 10^5^ cells/well. Cells were then transfected with SIRT1 siRNA (15 nM), or CHOP siRNA (30 nM; RiboBio) by use of Lipofectamine 3000 Transfection Reagent (Thermo Fisher Scientific) in accordance with the manufacturer's protocol. Scrambled siRNA was used as negative control siRNA (CTR siRNA). The protein expression of SIRT1 and CHOP in cells transfected with siRNA was examined by western blot analysis.

### 4.4 Arterial ring organ culture

Thoracic aortas were isolated from male SD rats euthanized with intraperitoneal injection of sodium pentobarbital (150 mg/kg). Human arteries were harvested from those patients who underwent lower limb amputation. Written informed consent was given by all patients prior to the inclusion. Human tissue use was approved by the Ethics Committee of Zhujiang hospital, Southern Medical University, China (Approval number: 2020‐KY‐045‐02), and this study was conducted in accordance with the Declaration of Helsinki. The characteristics of patients used in this study are included in Table S1. The aortas were cut into 2–3 mm rings and incubated with DMEM containing 10% FBS, 100 U/ml penicillin at 37°C in a humidified incubator within 5% CO_2_. Arterial segments were cultured with calcifying medium for 7 days to induce mineralization.

### 4.5 ALP activity assay

Alkaline phosphatase (ALP) activity was measured using alkaline phosphatase assay kit (Beyotime). Proteins were extracted by freeze‐thawing the cells in 0.1% Triton X‐100 in PBS. Protein concentration was determined using BCA Protein Assay (Thermo Fisher Scientific). Protein samples were mixed with p‐nitrophenylphosphate (p‐NPP) substrate and incubated for 10 min at 37°C. The reaction was terminated with 3 M NaOH. The absorbance was measured at 405 nm, and ALP activity was calculated as unit/mg protein.

### 4.6 SA‐β‐Gal Staining

Cellular senescence of VSMCs was determined using SA‐β‐Gal staining kit (Beyotime). Vascular smooth muscle cells were fixed with 4% formaldehyde for 15 min and washed with PBS for three times. Cells then were incubated with 1 ml of SA‐β‐Gal staining solution overnight at 37°C. Images were taken under an inverted microscope.

### 4.7 Determination of cell and tissue calcification

Alizarin red staining was used to determine the presence of cell and arterial calcification as described in our previous studies (Zhang et al., 2020). For cell staining, vascular smooth muscle cells were seeded in 35 mm dishes. After used medium was removed, cells were then fixed in 4% formaldehyde for 10 min and exposed to 2% alizarin red solution (pH 4.2) at room temperature for 5 min. Cells were rinsed with water to eliminate the excess dye, and images were taken under an inverted microscope. To quantitatively analyze the extent of calcification, alizarin red dye was incubated with 10% formic acid for 5 min, and the optical density at 405 nm was measured by a microplate reader. For arterial section staining, arterial segments fixed in 4% formaldehyde were embedded in paraffin. Arterial tissues were cut into 5 μm in thickness. Paraffin sections were dewaxed and incubated with 2% alizarin red solution. Images were captured under an inverted microscope. For the whole mount of aorta staining, aortic arteries were fixed in 95% ethanol for 24 h and then stained with 0.003% alizarin red solution in 1% potassium hydroxide overnight. The arterial tissues were then rinsed in 2% potassium hydroxide and photographed with an inverted microscope. Calcium content was determined using a commercial Ca assay kit (Leagene) in accordance with the manufacturer's protocol. In brief, cells or tissues were homogenized and the supernatant was separated by centrifugation. Two hundred microlitre of Methyl thymol blue (MTB) solution was mixed with 2.5 µl of samples and incubated for 10 min at room temperature. The absorbance was assessed at 610 nm using a microplate reader. BCA Protein Assay was used to measure total protein concentration. The relative calcium content normalized to the protein concentration of the lysates was expressed as μg/mg protein.

### 4.8 In vivo micro‐computed tomography

Micro‐computed tomography (Micro‐CT) was used to assess the extent of aortic calcification as previously described (Li et al., 2019). Briefly, animals were sacrificed by intraperitoneal injection of sodium pentobarbital (150 mg/kg). Rat aortas were then harvested and fixed with 4% formaldehyde at the end of experiment. Aortic samples were scanned using Micro‐CT (Siemens Inveon) at a resolution of 0.079 mm. Micro‐CT images were analyzed by the Inveon research workplace software (Siemens Inveon).

### 4.9 Quantitation of serum creatinine and fetuin‐A levels

Blood samples were collected from ophthalmic veins of rats 2 weeks after 5/6 nephrectomy surgery. The plasma was centrifuged, and the upper layer of serum was collected. Levels of plasma creatinine in rats were measured by a colorimetric method using a BioAssay Systems Creatinine Assay Kit (BioAssay Systems). Serum levels of fetuin‐A in rats were determined using rat ELISA kits (Elabscience).

### 4.10 Western blot analysis

Vascular smooth muscle cells and arterial tissues were homogenized with RIPA buffer containing proteinase and phosphatase inhibitors. The protein concentrations were quantified with Pierce™ BCA Protein Assay Kit (Thermo Fisher Scientific) according to the manufacturer's guidelines. Proteins separated by SDS‐PAGE were transferred to PVDF membranes, followed by being incubated with 5% milk overnight at 4°C. Membranes were immunoblotted using specific primary antibodies: anti‐BMP2 (1:1000, ab214821; Abcam), anti‐Runx2 (1:1000, 12556S; Cell Signaling Technology), anti‐SIRT1 (1:1000, 9475S; Cell Signaling Technology), anti‐CHOP (1:1000, 15204–1‐AP; Proteintech), anti‐ATF4 (1:1000, 10835–1‐AP; Proteintech), anti‐p21 (1:1000, ab109199; Abcam), and anti‐β‐actin (1:1000, 4970S; Cell Signaling Technology). Membranes were rinsed with PBST for three times, and then incubated with HRP‐conjugated secondary antibodies. The blots were visualized by the imaging system (Amersham Imager 600) and quantified using Image J software.

### 4.11 Statistical analysis

All results were presented as Mean ± SEM. Differences among more than two groups were compared using one‐way ANOVA followed by Tukey's HSD test or Tamhane's T2 test. All statistical analyses were performed by use of software SPSS 20.0 and *p* < 0.05 was accepted as statistically significant.

## CONFLICT OF INTEREST

The authors declare no conflict of interest.

## AUTHOR CONTRIBUTIONS

X.L. performed experiments, analyzed the data and wrote the draft. A.C. and Q.L. performed experiments and analyzed the data. X.Y., Q.D., M.F., S.W., Y.L., Y.Y., Z.L., Y.C., J.S.O., and P.Y. performed experiments. L.L. and J.Y. designed the experiments, analyzed the data, and revised the manuscript.

## Supporting information

Supplementary Material

Supplementary Material

## Data Availability

The data that supports the findings of this study are available in the [Link], [Link] of this article and from the corresponding author upon reasonable request.
