# Supplementary material for: Spermidine inhibits vascular calcification in chronic kidney disease through modulation of SIRT1 signaling pathway
Source: Aging Cell. 2021 May 9;20(6):e13377. doi: 10.1111/acel.13377 (PMC8208796; doi:10.1111/acel.13377)
Supplement: Supplementary file 1 — Supplementary Material [file ACEL-20-e13377-s001.docx]

**Spermidine inhibits vascular calcification in chronic kidney disease through modulation of SIRT1 signaling pathway**

Xiaoyu Liu^1#^, An Chen^1#^, Qingchun Liang^2#^, Xiulin Yang^1^, Qianqian Dong^1^, Mingwei Fu^1^, Siyi Wang^1^, Yining Li^1^, Yuanzhi Ye^1^, Zirong Lan^1^, Yanting Chen^3^, Jing-Song Ou^4^, Pingzhen Yang^1^, Lihe Lu^3*^, Jianyun Yan^1*^

^1^Department of Cardiology, Laboratory of Heart Center, Heart Center, Zhujiang Hospital, Southern Medical University; Guangdong Provincial Biomedical Engineering Technology Research Center for Cardiovascular Disease; Sino-Japanese Cooperation Platform for Translational Research in Heart Failure, Guangzhou 510280, China

^2^Department of Anesthesiology, The Third Affiliated Hospital, Southern Medical University, Guangzhou 510665, China

^3^Department of Pathophysiolgy, Zhongshan School of Medicine, Sun Yat-Sen University, Guangzhou 510080, China

^4^Division of Cardiac Surgery, The First Affiliated Hospital, Sun Yat-Sen University, Guangzhou 510080, China

**Running title:** Spermidine inhibits vascular calcification

^#^These authors contributed equally to this work.

***Corresponding authors:** Jianyun Yan or Lihe Lu

Jianyun Yan, PhD, Department of Cardiology, Heart Center, Zhujiang Hospital, Southern Medical University, 253 Industrial Avenue, Guangzhou, 510280, China

E-mail: yanjy790@smu.edu.cn

Tel: +86 20 62782264

Lihe Lu, PhD, Department of Pathophysiolgy, Zhongshan School of Medicine, Sun Yat-Sen University, 74 Zhongshan Er Road, Guangzhou, 510080, China

E-mail: lulihe@mail.sysu.edu.cn

**Supplementary data**

**
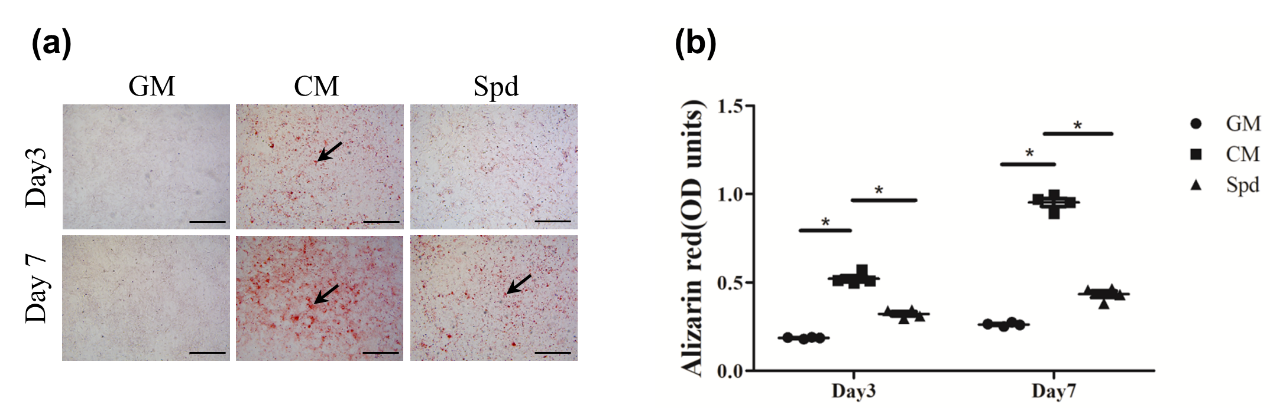
**

**Supplementary Figure S1. Spd inhibits calcification of rat vascular smooth muscle cells.** Rat vascular smooth muscle cells were incubated with growth medium (GM), or calcifying medium (CM) with or without Spd (1.0 μM) for 3 or 7 days (n=4). (a) Alizarin red staining of cells was performed over one-week time course. Representative images showing cells stained with alizarin red solution. Scale bar=500 µm. (b) Quantitative analysis of alizarin red staining by a microplate reader. *P<0.01. One-way ANOVA, Tukey’s HSD post hoc test.


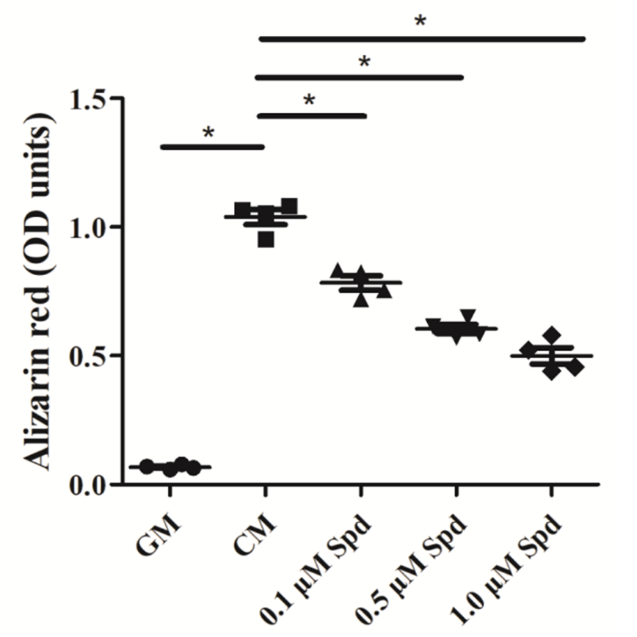


**Supplementary Figure S2. Spd inhibits calcification of human vascular smooth muscle cells.** Human vascular smooth muscle cells were incubated with growth medium (GM), or calcifying medium (CM) with or without different concentrations of Spd (0.1 μM, 0.5 μM, 1.0 μM) for 7 days (n=4). Quantitative analysis of alizarin red staining by a microplate reader. *P<0.01. Tamhane T2 test.


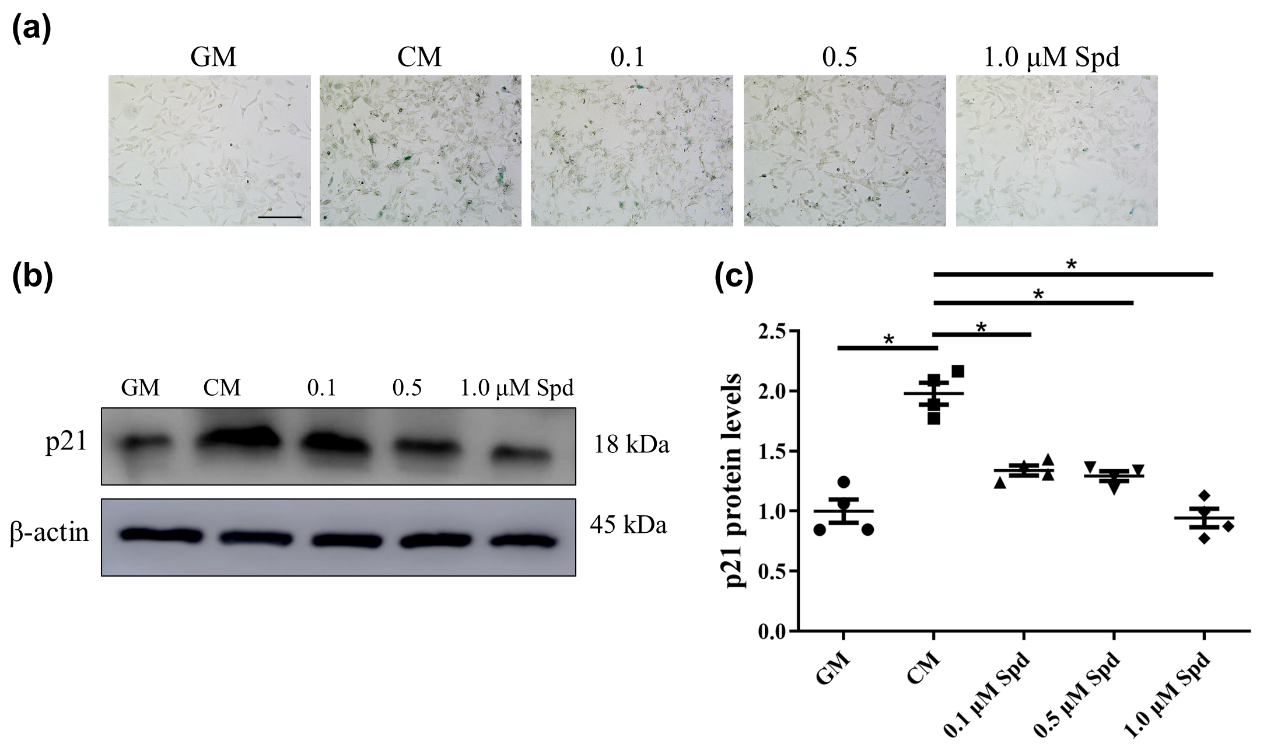


**Supplementary Figure S3. Spd inhibits rat vascular smooth muscle cell senescence.** Rat vascular smooth muscle cells were incubated with growth medium (GM), or calcifying medium (CM) with or without Spd (0.1 μM, 0.5 μM, 1.0 μM) for 7 days (n=4). (a) SA-β-Gal staining of cells was performed. Representative images showing cells stained with SA-β-Gal solution. Scale bar=250 µm. (b) Western blot analysis of p21 expression. (c) Quantitative analysis of p21 expression. *P<0.01. One-way ANOVA, Tukey’s HSD post hoc test.

**
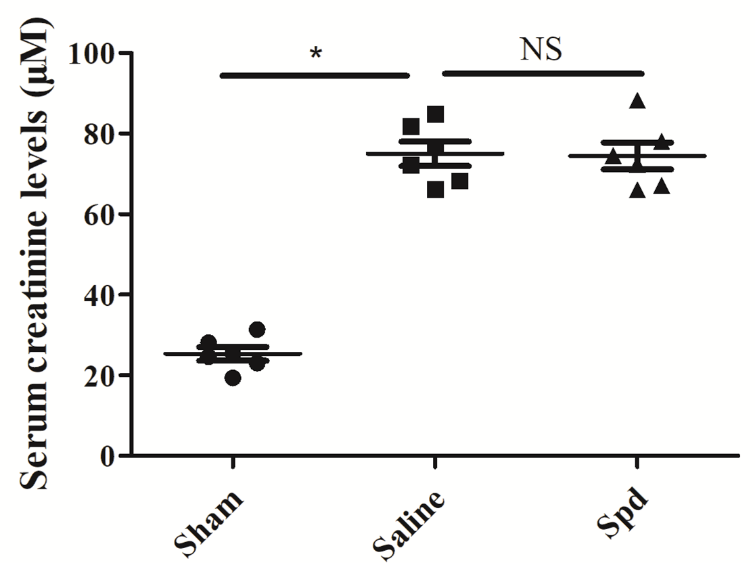
**

**Supplementary Figure S4. Serum creatinine levels were increased in rats with chronic kidney disease.** Rats were randomly assigned into sham group, CKD model (saline) group, and Spd group. All rats except sham group were subjected to surgery. Rat CKD model was generated by 5/6 nephrectomy method (n=6). Serum creatinine levels were measured two weeks after surgery. *P<0.01. One-way ANOVA, Tukey’s HSD post hoc test. NS= no significance. CKD= chronic kidney disease.


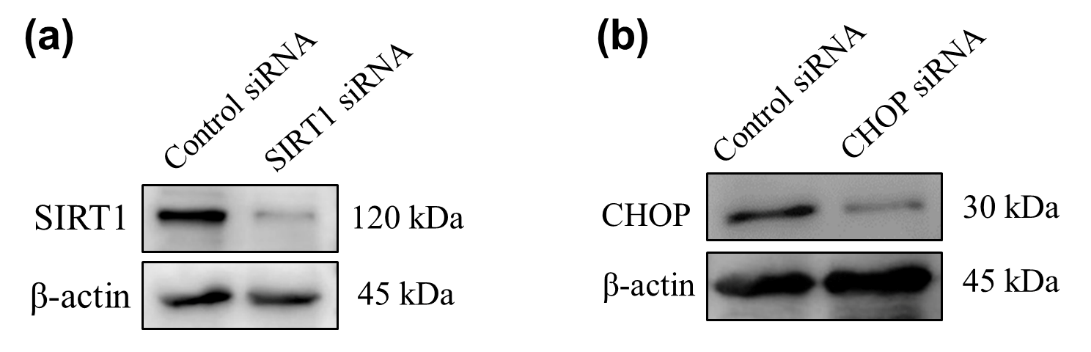


**Supplementary Figure S5. SIRT and CHOP expression was down-regulated in rat** **vascular smooth muscle cells transfected with SIRT1 siRNA or CHOP siRNA.** Rat vascular smooth muscle cells were transfected with Control siRNA, SIRT1 siRNA or CHOP siRNA in growth medium for 2 days. Western blotting was performed to analyze SIRT1 and CHOP expression. Representative western blots for SIRT1 (a) and CHOP (b).

**Supplementary Table**

**Supplementary Table S1. Characteristics of patients used in this study for human** **arterial ring organ culture.** Tibial or femoral artery samples were obtained from patients subjected to a lower limb amputation.

| ID | Age | Sex | Artery samples |
| --- | --- | --- | --- |
| 1 | 50 | Male | Tibial artery |
| 2 | 67 | Male | Femoral artery |
| 3 | 75 | Female | Tibial artery |
| 4 | 70 | Female | Tibial artery |
