# Supplementary material for: Spermidine inhibits vascular calcification in chronic kidney disease through modulation of SIRT1 signaling pathway
Source: Aging Cell. 2021 May 9;20(6):e13377. doi: 10.1111/acel.13377 (PMC8208796; doi:10.1111/acel.13377)

Full unedited gel for Figure 1e

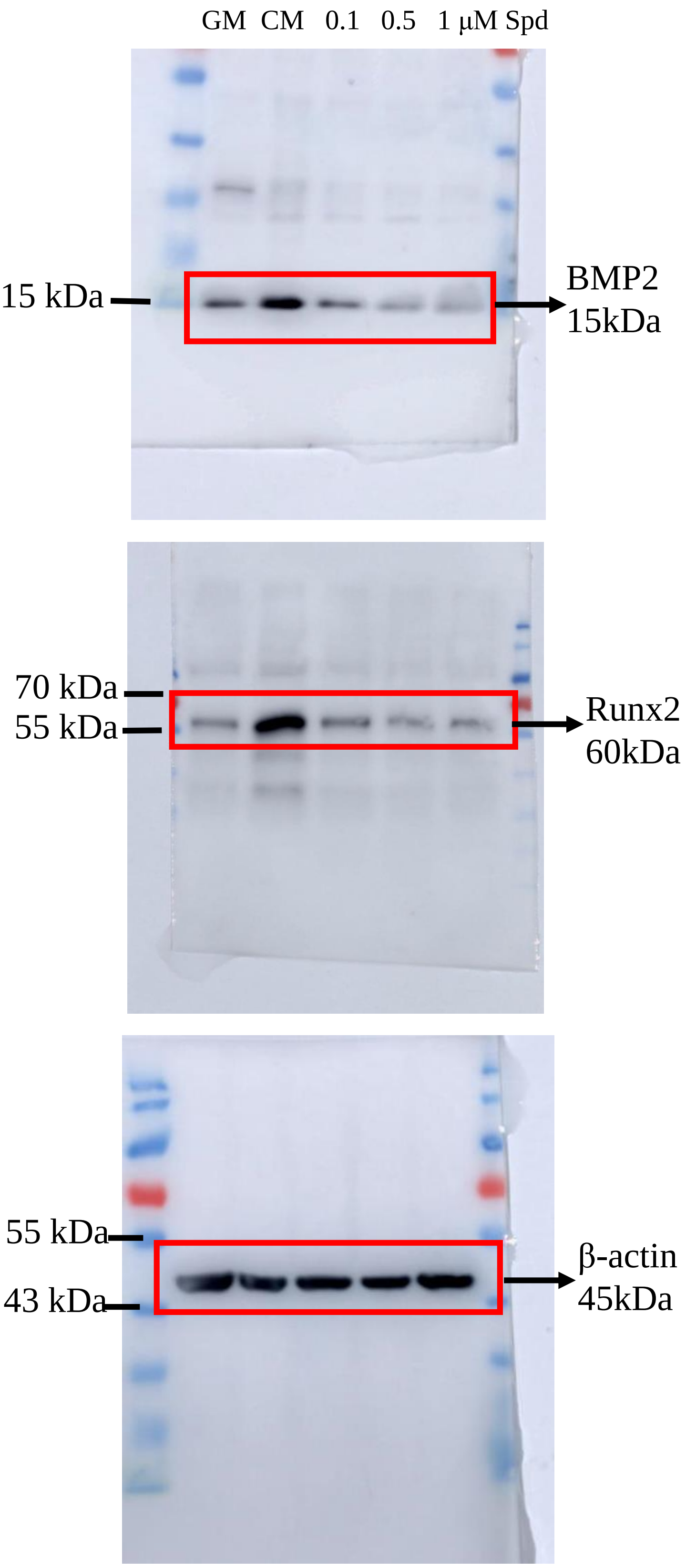

Full unedited gel for Figure 1i

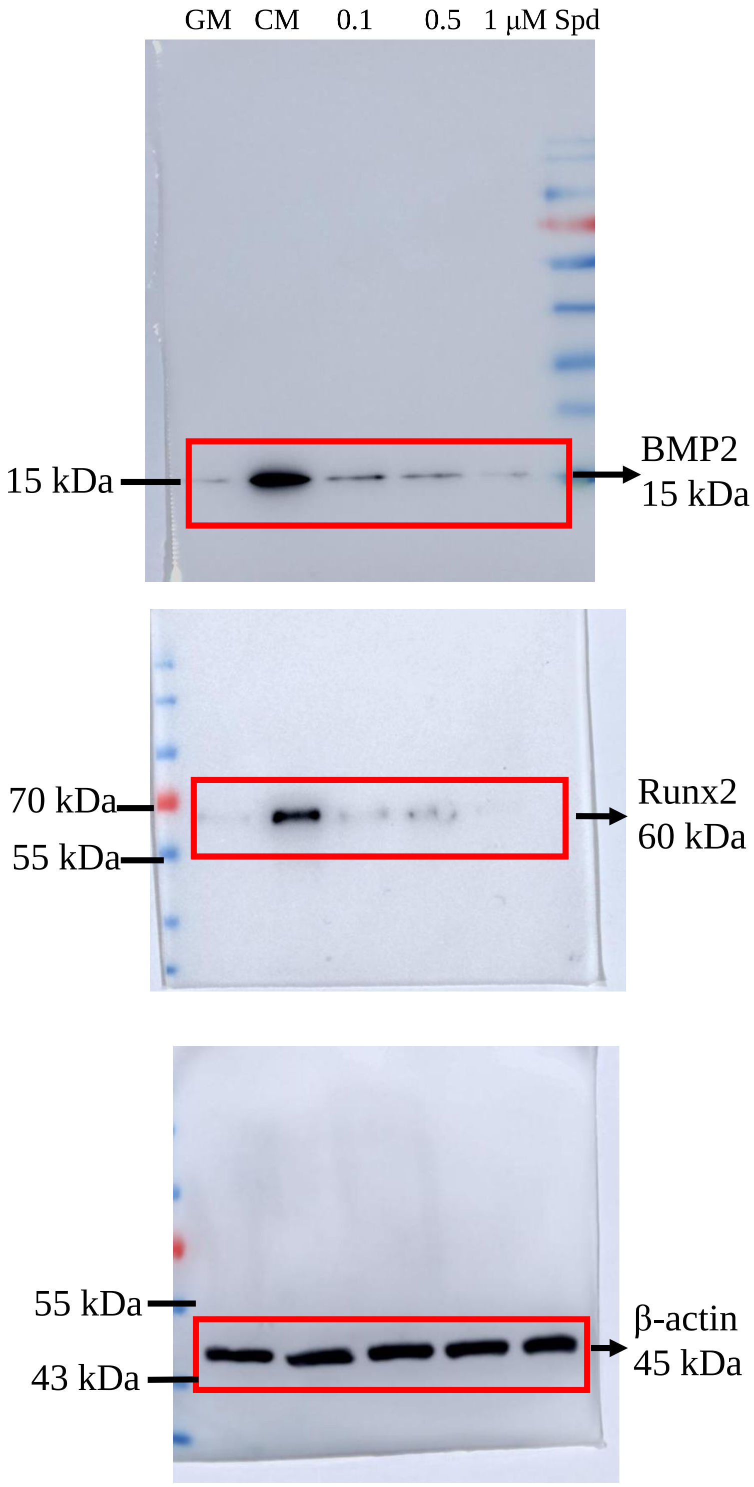

Full unedited gel for Figure 3e

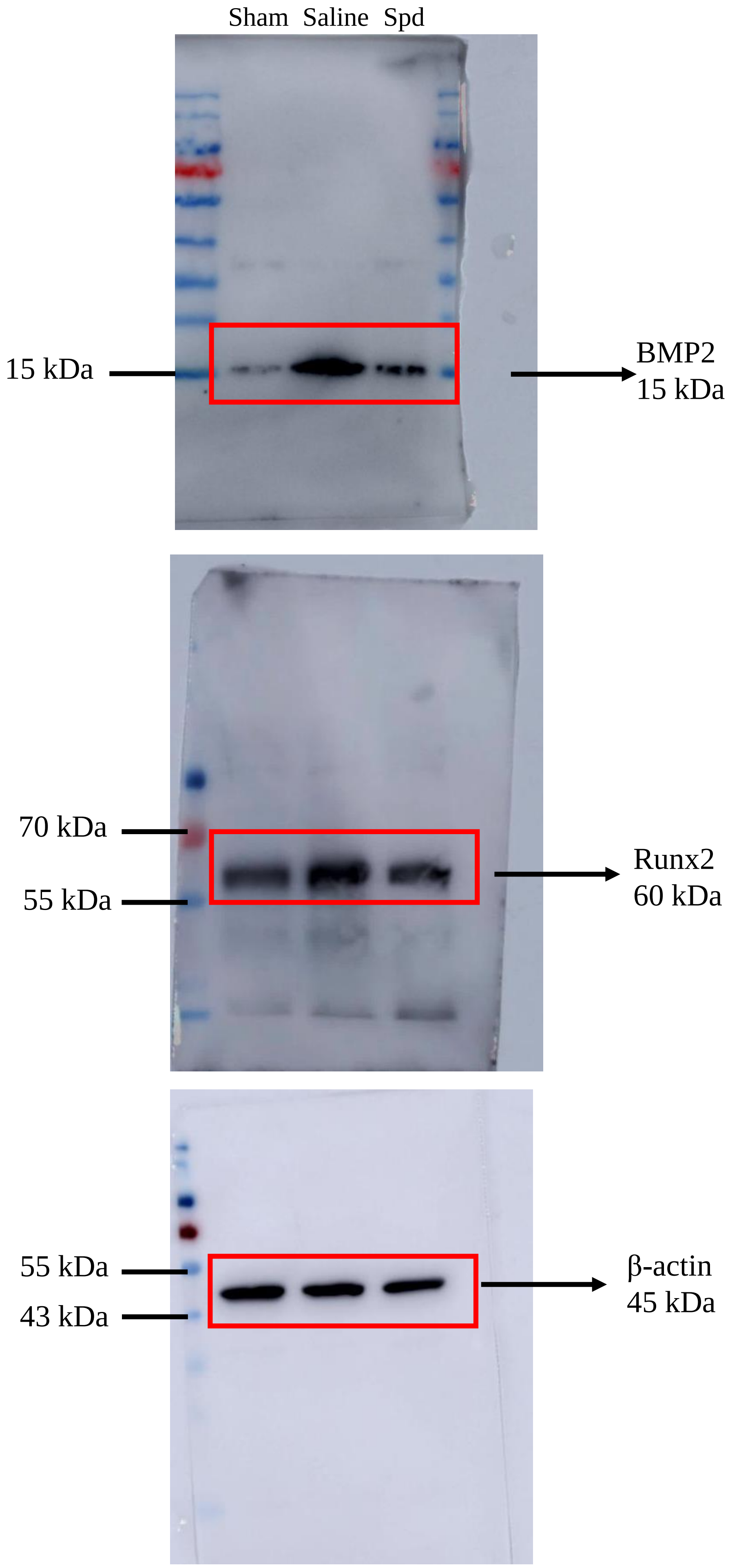

Full unedited gel for Figure 4a

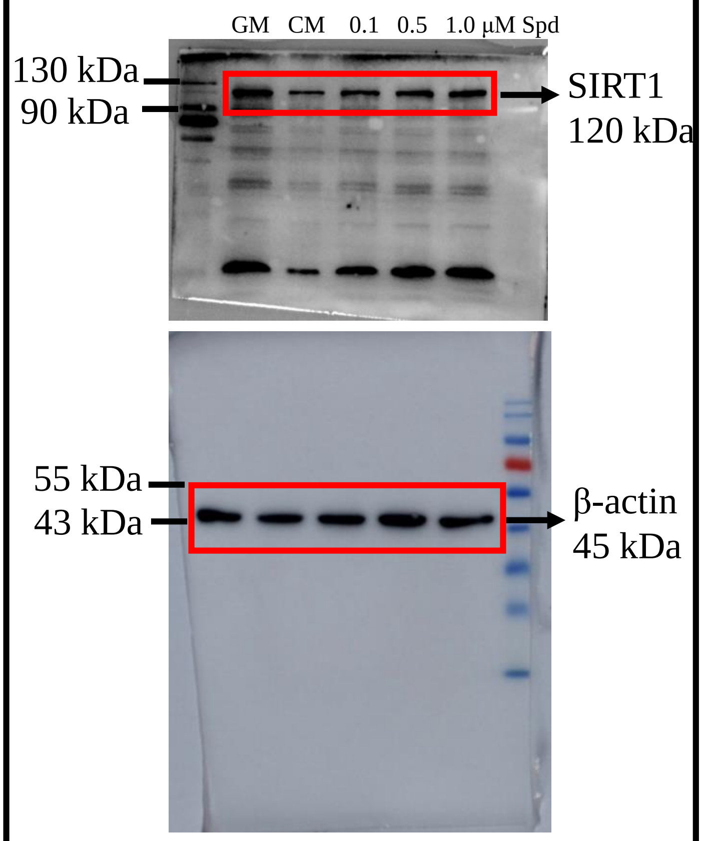

Full unedited gel for Figure 4f

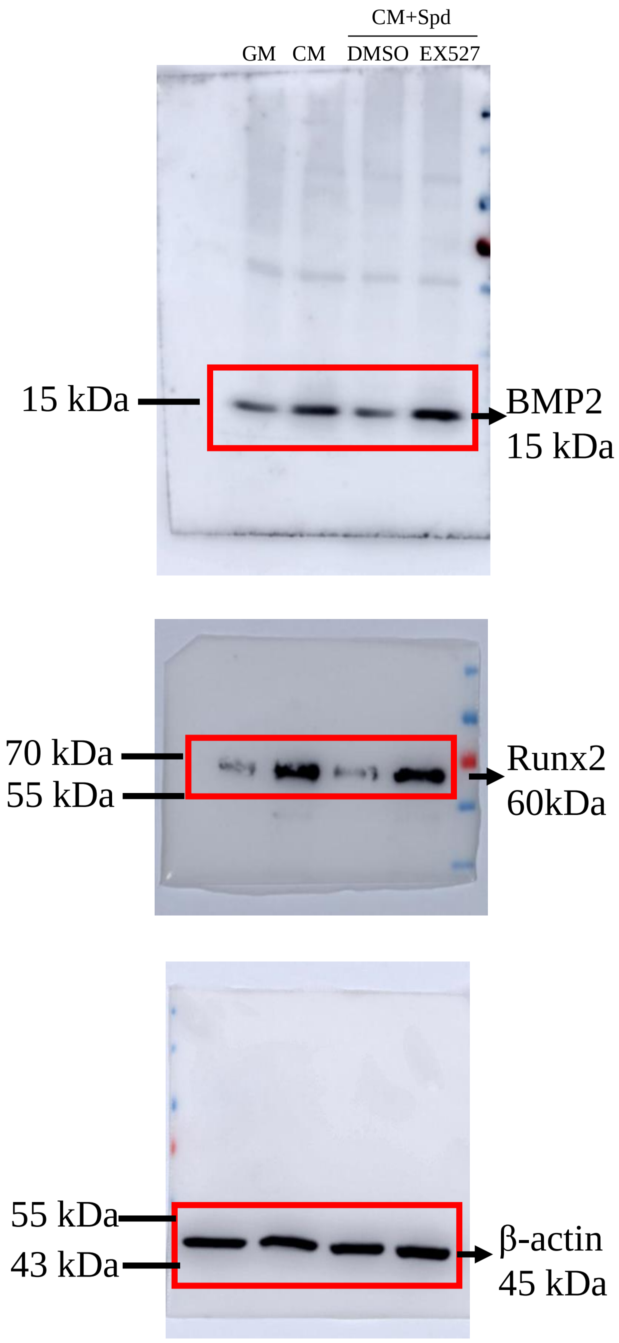

Full unedited gel for Figure 5d

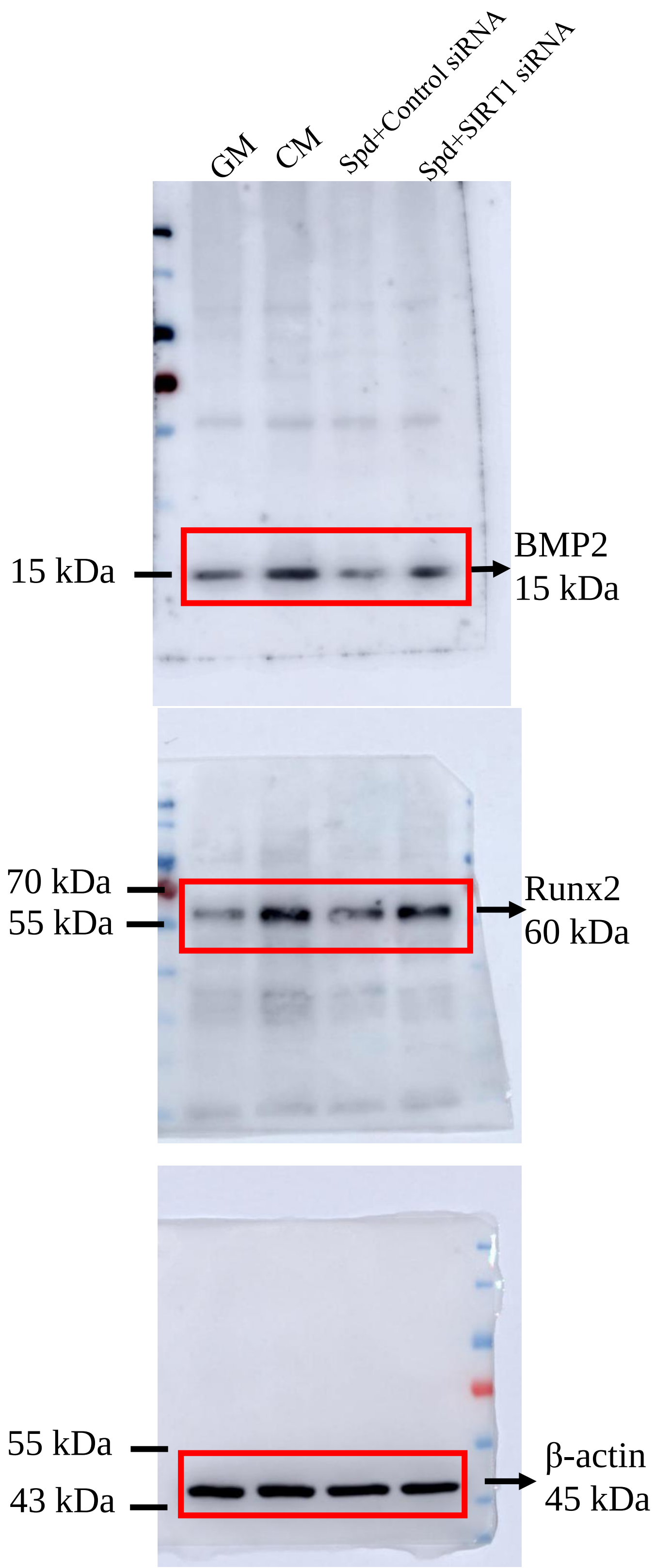

Full unedited gel for Figure 6e

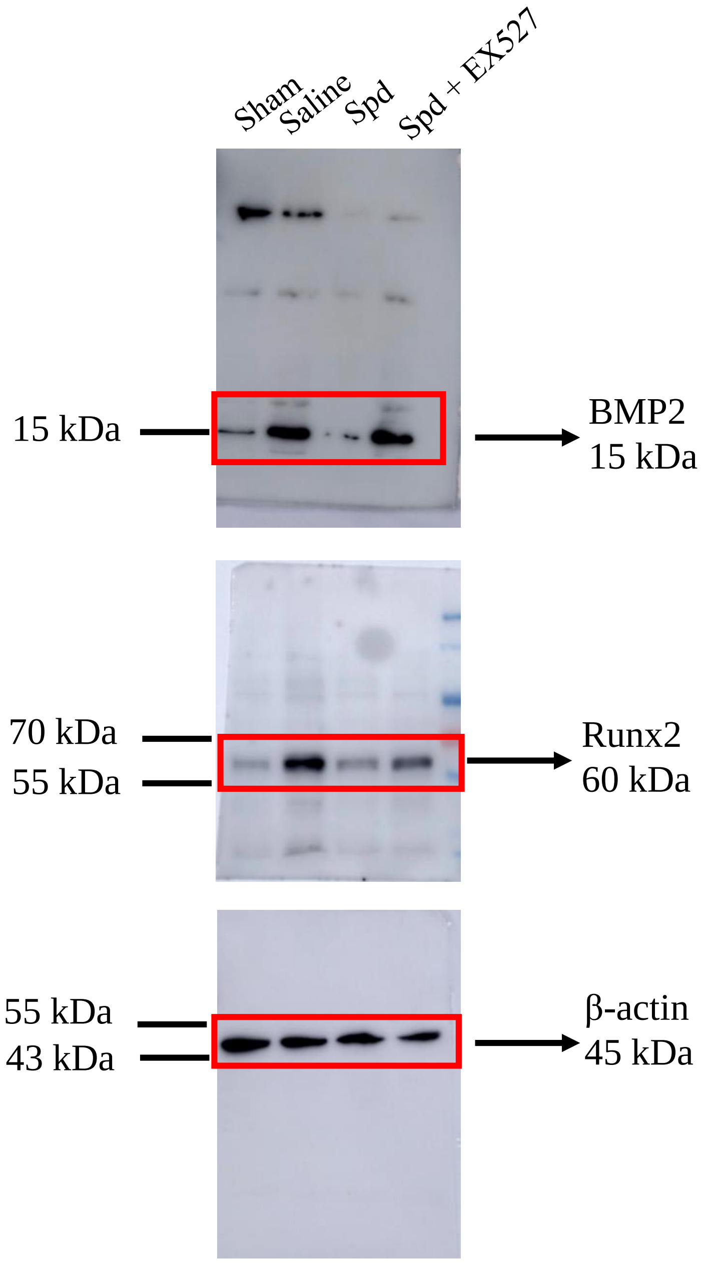

Full unedited gel for Figure 7a

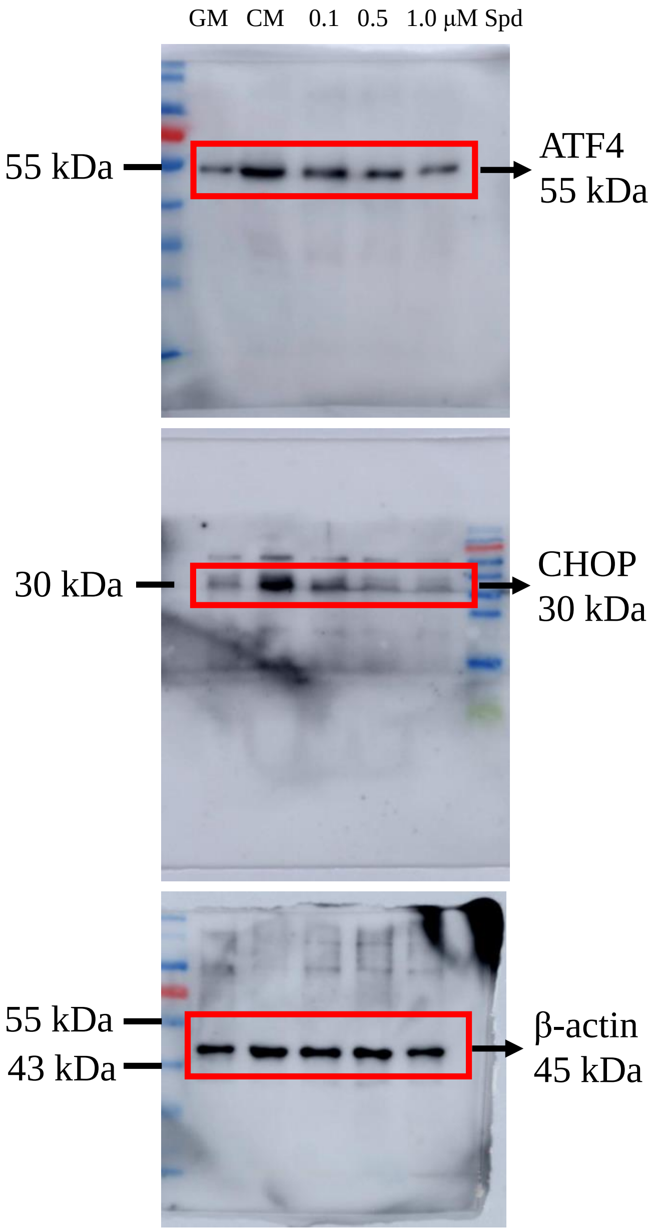

Full unedited gel for Figure 7c

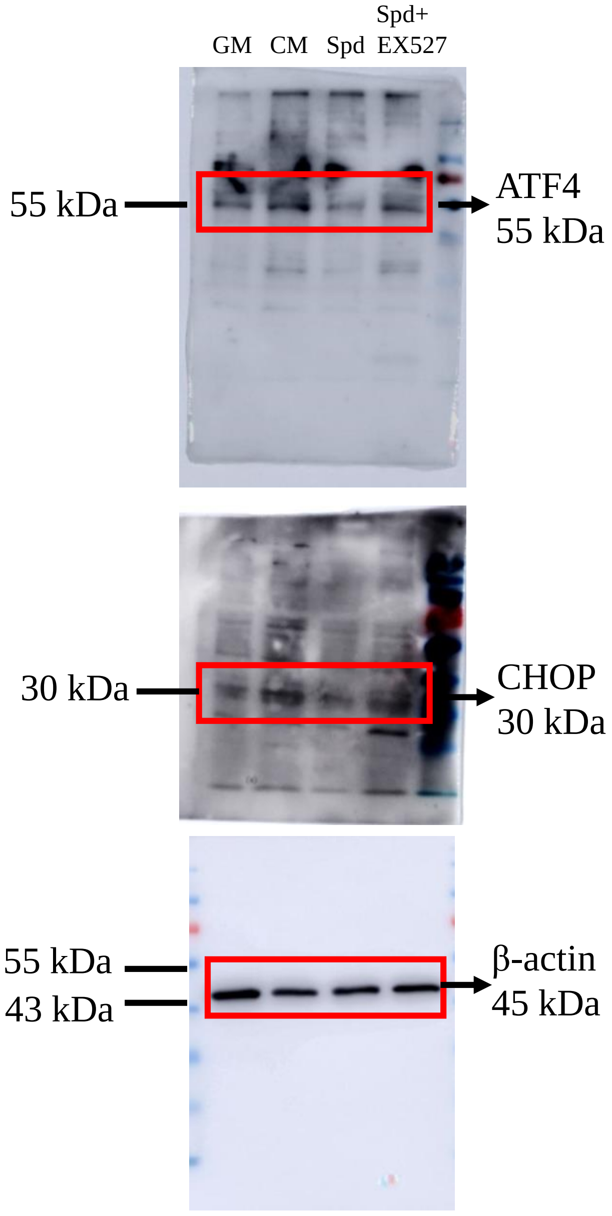

Full unedited gel for Supplemental Figure S3b

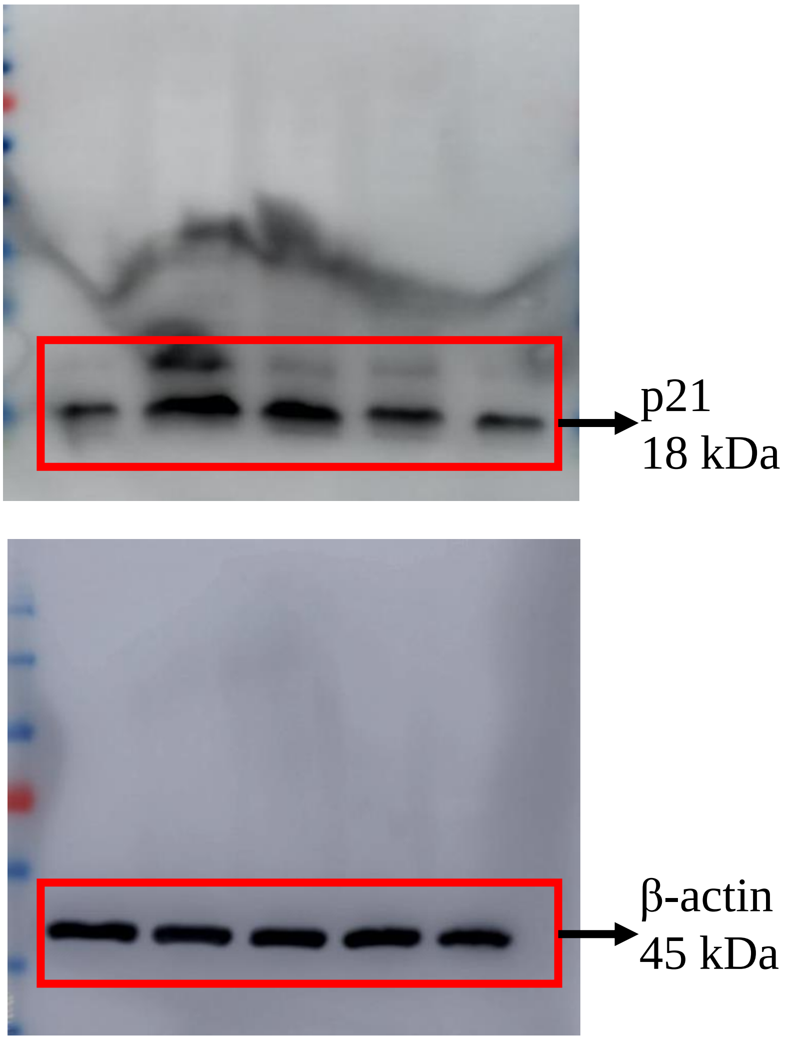

Full unedited gel for Supplementary Figure S5a

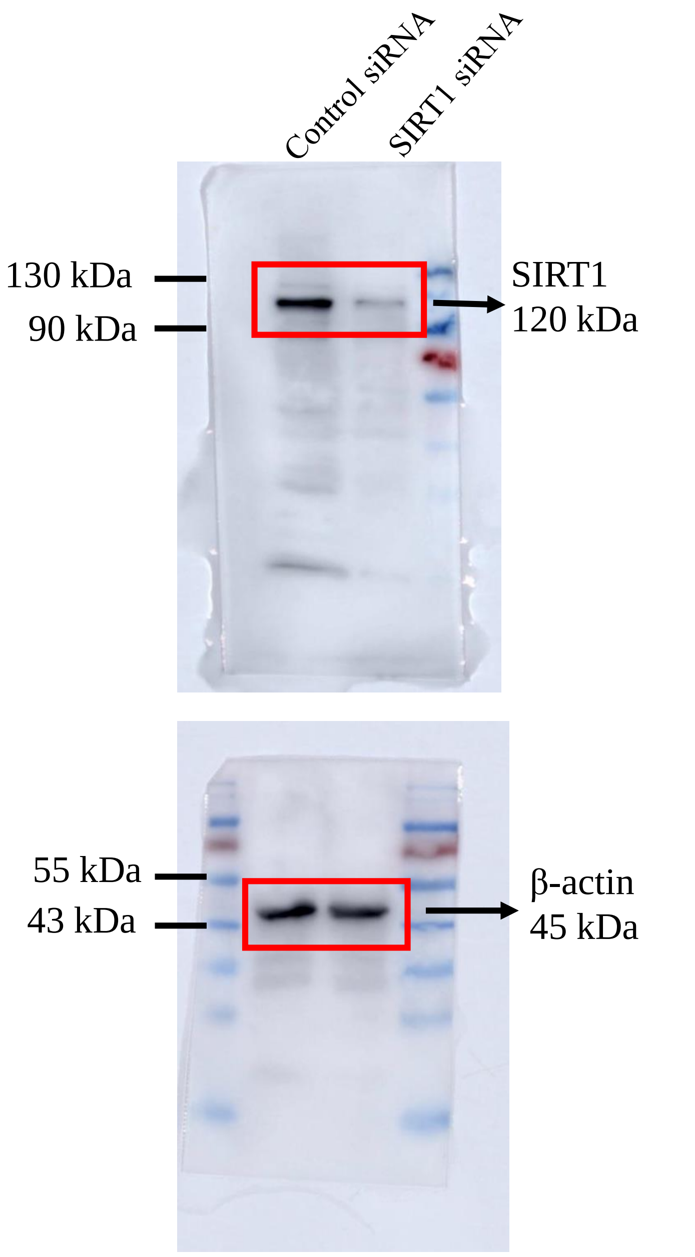

Full unedited gel for Supplementary Figure S5b

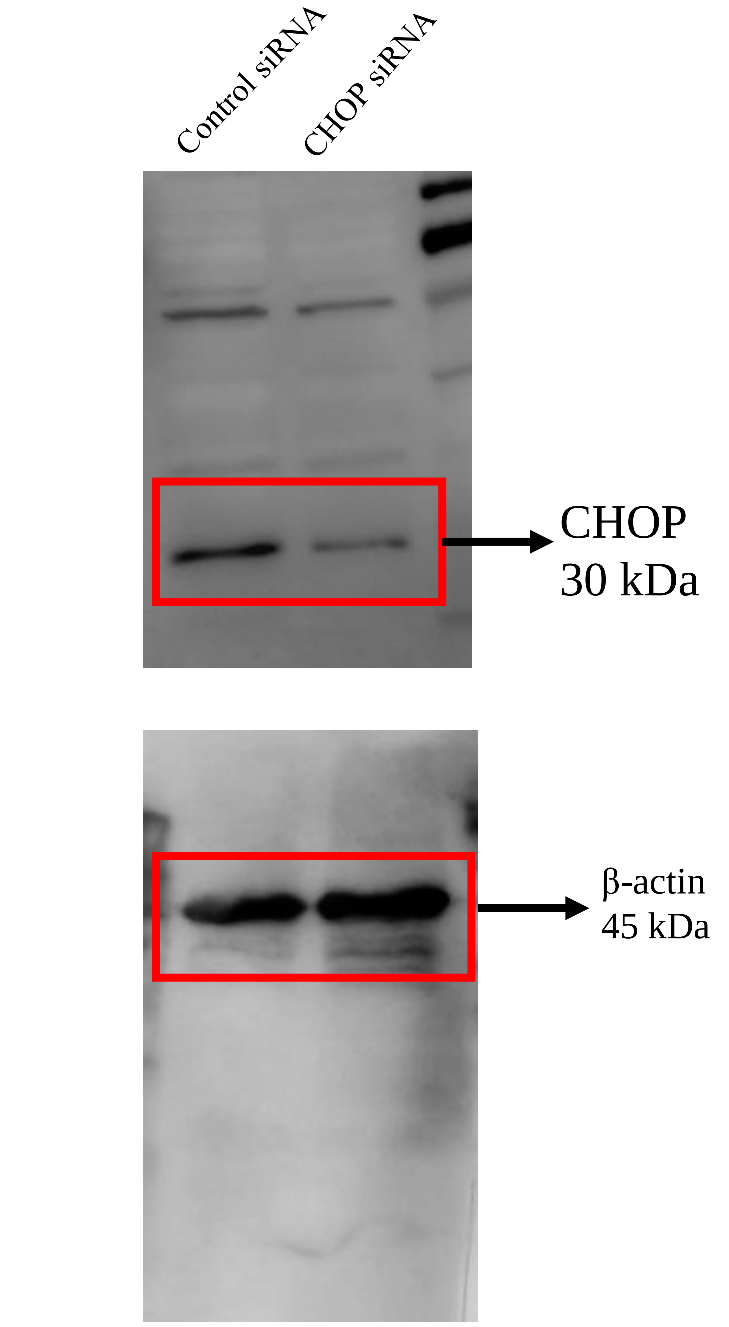

Supplement: Supplementary file 2 — Supplementary Material [file ACEL-20-e13377-s002.pdf]
